# Conditional Climate–Cost Trade-Offs and Transport Break-Even Conditions for Waste Flexible-Film Recycling Under Feedstock Quality Constraints

**DOI:** 10.3390/polym18212646

**Published:** 2026-10-25

**Authors:** Chaojie Yu, Junqing Xu, Xichao Hu, Haochen Zhu, Guangming Li

**Affiliations:** 1 College of Environmental Science and Engineering, Tongji University, Shanghai 200092, China; 2030524@tongji.edu.cn (C.Y.); xjq0512@tongji.edu.cn (J.X.); haochen_zhu@tongji.edu.cn (H.Z.); 2 Shanghai Tianqiang Environmental Technology Co., Ltd., Shanghai 201415, China; 19117171706@163.com

**Keywords:** waste flexible film, feedstock quality, mechanical recycling, climate impact, covered costs, transport break-even, constrained allocation

## Abstract

Feedstock quality and unequal process services complicate comparisons of waste flexible-film recycling routes. We linked records from 52 recyclers and 166 flows with 60 composition observations and separate polyethylene (PE) pelletization and mixed-feed (MIX) molding records. PE scenarios distinguished fixed feed from equal retained pellet output; MIX scenarios compared treatment of the same feed without assuming equivalent product function. Twelve PE batches yielded 38,226 kg of pellets from 40,796 kg of feed, with pooled electricity use of 439.8 kWh/t pellets. Industrial dry-cleaning electricity was treated as a threshold variable. At the MIX-06 reference setting, the covered climate impact of the extrusion reference exceeded molding by 23.5 kg CO_2_-eq/t feed; this difference ranged from −58.2 to 129.7 kg CO_2_-eq/t across engineering scenarios. Additional relative molding transport removed the climate and L2 cost margins at 150.5 and 34.0 km. L2 includes priced direct inputs, labor, maintenance and annualized equipment costs. Monthly throughput weighting yielded a candidate recorded flow interval of 2728.7–6605.2 t before deployment constraints. Route rankings depend on specified services, quality, capacity and feed supply. Unquantified services limit industrial interpretation, and unresolved resale links prevent interpreting regional endpoints as deduplicated physical savings.

## 1. Introduction

Feedstock composition and contamination shape the mechanical recycling of waste flexible film. Waste origin, adhering contaminants, and sorting conditions determine pretreatment requirements [1], while thermomechanical degradation and polymer incompatibility restrict recyclate properties and applications [2,3]. Multilayer structures complicate both component separation and joint processing [4]. Identifying the polymers on the inner and outer packaging surfaces does not establish the complete layer structure [5]. Ink binders and pigments in printed films can also affect processability, mechanical properties, and appearance [6]. Recycling route assessment therefore requires information on both resin and contaminant composition, together with explicit quality requirements for the recycled product.

Studies of flexible-plastic recycling have linked material flow analysis to process yields, polymer grades, and transparency [7]. Others have assessed how improvements in sorting, washing, and extrusion affect material properties and economic value [8]. Classifying waste streams by recycling difficulty also helps identify materials suitable for mechanical recycling [9]. Broader assessments of recycling quality consider virgin material substitution potential, time in use, and environmental impacts [10], while distinguishing material, component, and product levels [11]. Evidence at these levels addresses different questions. Processing degradation and impurities affect recycled polyethylene (PE) properties [12,13], and compositional fractionation can quantify polypropylene (PP) cross-contamination in PE [14]. Washing, melt compounding, processing conditions, and antioxidants also influence material stability and product performance [15,16]. Feedstock classification, process performance, and product suitability must therefore be distinguished when comparing recycling routes.

Recent flexible-film experiments also link sorting quality to residual inclusions and mechanical performance, while recognizing that washing and melt filtration contribute to differences between recyclates [17].

Climate and economic comparisons depend on the service assigned to each route. Assessments per unit of waste treated and per unit of recycled product use different functional units and system boundaries [18]. Credits for virgin material displacement additionally require evidence of technical and market substitutability [19,20,21]. Contamination and substitution ratios affect recycling assessments [22,23]. Additional material needed to maintain film functionality can also offset climate advantages calculated per unit mass of pellets [24]. Pretreatment and industrial recycling inventories show why washing, drying, pelletization, and wastewater treatment must be accounted for within consistent boundaries [25,26]. Route rankings can differ across energy systems and environmental indicators [27,28]. Techno-economic studies jointly consider yield, energy demand, material quality, and product value [29,30,31], while equipment-specific extrusion measurements provide inputs for process scenarios [32]. Accordingly, this study separates the PE product-output comparison from the mixed-feed (MIX) treatment comparison, then evaluates how transport and costs modify the process-level differences.

Recent studies further clarify these comparison requirements. A basket-of-functions analysis accounts explicitly for the different services provided by high-quality recycling and downcycling [33]. A film-recycling life cycle assessment (LCA) examines how transport allocation, processing scale and product design affect the comparison [34]. Joint life cycle assessment and the costing of flexible packaging also retains material losses and limits on recyclate incorporation into new packaging [35]. These studies support specifying the treatment and output services before interpreting process, transport and cost differences.

Regional analysis is further complicated by differences in the level of detail among available datasets. Polymer- and application-resolved material flow analysis describes plastic production, trade, and waste management [36]. Combining detailed flows with feedstock-dependent process inventories allows evaluation of alternative routing strategies [37]. System-level studies also show that industrial flow information and statistical coverage affect the completeness of material accounts [38,39]. Enterprise records, composition samples and transport records describe quantities, quality at particular sampling times, and destinations, respectively. Data reconciliation and uncertainty methods provide a basis for distinguishing these sources of information [40,41].

Previous studies have linked flexible-plastic composition to processing performance and distinguished waste-treatment from recycled-product comparisons [7,18,19]. However, enterprise-level quality observations do not identify the quality of individual recorded destination flows. The allocation model preserves recorded flow totals and the enterprise quality marginals selected by each annual proxy. It then calculates minimum and maximum route differences across admissible quality–flow assignments. Annual-proxy variation and within-enterprise allocation are evaluated separately, without assigning an unobserved composition to an individual flow [37,41].

We combine the quality–flow allocation model with route-specific service definitions and constraints on deployment. PE scenarios distinguish fixed feed from equal retained pellet output, accounting for recovery, assumed retained-output shares, capacity and additional feed. MIX scenarios compare treatment of the same feed while retaining separate product quantities. Transport and unquantified service thresholds identify conditions that change the covered route ranking. The analysis draws on the team’s pulsed gas–solid cleaning platform [42], with laboratory observations, production records and transferred inventories kept at their respective evidence levels.

## 2. Materials and Methods

### 2.1. Study Scope and Data Sources

The study uses records from recycling enterprises in Shanghai and their recorded destinations. Monthly ledgers span September 2020–August 2021, with composition observations in October and December 2020 and March and June 2021. MIX molding records date from February 2023 and PE pelletization records from November 2024. The 2020–2021 records describe the handling network and annual quality proxies; the later production records provide inputs for separately documented process services. Applying those services to the recorded network assumes their transferability and does not reconstruct historical plant performance. The 2026 designation identifies the economic comparison scenario, rather than a common measurement or verified quotation year. Table 1 and Table S1 list dataset dates, sources, analytical uses and transfer assumptions. Evidence labels distinguish direct measurement, enterprise-derived record, transferred inventory, engineering estimate and analyst-defined scenario; the labels describe provenance without implying independent validation.

A companion paper [43] analyzes laboratory cleaning subsamples from the same twelve material-source batches as the separately documented PE production batches. The laboratory subsamples did not enter the production line. The manuscripts share material origins, but the laboratory records neither document a continuous cleaning–pelletization trial nor provide industrial cleaning electricity measurements. Table S2 identifies the source relationships and variables used in each manuscript.

**Figure 1:**
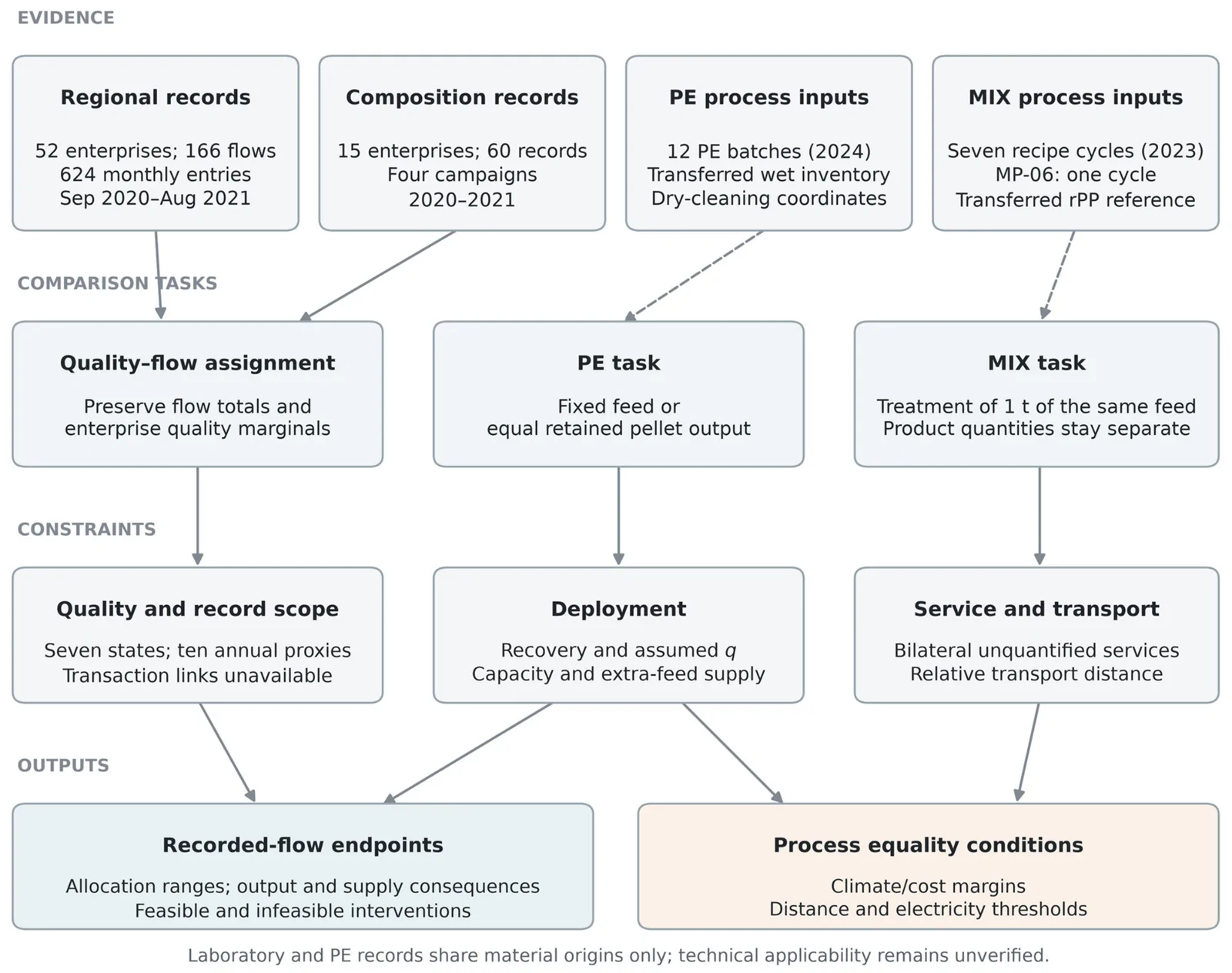
Evidence, comparison tasks, constraints and conditional outputs. PE: polyethylene. MIX: mixed feed. rPP: recycled polypropylene. *q*: assumed retained-output share. Network and composition records define flow quantities and quality proxies; separate production records and transferred inventories define process inputs. PE comparisons distinguish fixed feed from equal retained pellet output, while MIX retains the same-feed treatment task. Dashed connections denote conditional transfer or shared material origins. Technical applicability and transaction-level intervention links remain unverified. Quality allocation, assumed retained-output shares, capacity, additional supply and transport determine the calculated ranges and thresholds. Background shading groups the workflow blocks and distinguishes the two output types.

Incoming waste carries no burden from preceding virgin polymer production under the cut-off approach. The boundary covers the specified treatment or pellet-output service, without virgin material displacement credits. Climate subtotals include only activity data matched to background factors. Unmatched residue treatment, media regeneration and other route-specific services remain explicit unknown contributions. Foreground equipment manufacture is excluded from both routes, while infrastructure embedded in background datasets is retained. Product lifetime and food-contact suitability are not evaluated. Figure 1 summarizes the data sources, comparison tasks and constraints.

### 2.2. Feedstock Composition, Quality States, and Route Eligibility

The retained composition dataset comprises 60 compiled wet-mass, dry-mass and component-mass records from four campaigns at fifteen enterprises. According to the reported procedure, incoming material information and portable infrared spectroscopy (IR2200; Optosky Photonics Inc., Xiamen, China) were used to assign plastic categories. Paper, mineral matter and oil-associated material were recorded as operationally separated categories. Other impurity mass was calculated by subtracting the seven listed component masses from dry mass; it was not measured independently. The supplied archive does not contain detailed weighing sheets, sample-specific spectral records, regional-sample drying conditions or a validated oil-specific assay. Table S3 and Sampling_Characterization_Register.csv identify available evidence and unavailable sampling, identification and confirmation fields for each record. Regional sample weighing used a PTY-2003 precision balance (Huazhi (Fujian) Electronic Technology Co., Ltd., Putian, China). Wet-basis moisture and dry-basis component fractions were calculated from the recorded masses:

(1) $$w\;=\;\frac{m_{w}\;-\;m_{d}}{m_{w}}\;;\;x_{i}\;=\;\frac{m_{i}}{m_{d}}$$

In Equation (1), *w* is wet-basis moisture, *x_i_* is the dry-mass fraction of component *i*, and *m*_w_, *m*_d_ and *m_i_* are wet, dry and component masses. Principal symbols and units are listed in Table S4. Non-plastic impurities comprise the recorded paper, mineral, oil-associated and derived other-impurity categories. PE plus PP defines the polyolefin fraction; polyethylene terephthalate (PET) plus other plastics defines the other-polymer fraction. Classification uses retained masses before percentage display rounding.

The joint criteria in Table 2 define record-based classes. Classes I and II are subdivided into PE and PP; unmatched, multiply matched or insufficiently characterized records retain classification state U. A recorded zero denotes no assigned mass in that category and provides no analytical detection limit. Arithmetic closure cannot validate composition measurements, particularly because other impurities are calculated by difference. Technical applicability is assessed separately: material with unconfirmed contamination removal, equipment compatibility or target-product suitability remains unresolved in that layer. The PE equipment requires moisture ≤1% and particle sizes of 5–15 mm; the MIX equipment requires PET ≤5%. Table S5 records these inlet conditions and the additional service and product requirements for each candidate route. Classification-rule sensitivity is summarized in Table S6.

The MIX comparison uses the MIX-06 feedstock: 46.1% PE, 32.8% PP, 3.5% PET and 15.6% paper on a dry-mass basis, with a 2.0% unassigned remainder. The remainder is calculated by difference and has no established composition. Moisture of 2.02% and ash of 5.96% were measured separately; neither is added to the dry-component total. Composition samples and molding feed came from the same homogenized incoming batch. Sample drying for analysis was not the production pretreatment. Portable infrared identification used an IR2200-RT instrument (Optosky Photonics Inc., Xiamen, China).

Classification criteria and equipment inlet requirements were applied separately. PET ≤5% was an equipment requirement in this case, whereas other polymers <7% was a Class III feedstock criterion. Section 2.4 identifies the materials used for cut-specimen testing and the separate pallet-load tests.

### 2.3. Linking Regional Material Flows and Composition Samples

The retained registry contains 64 entities, of which 52 supplied twelve monthly entries each. Their 624 enterprise–month records reconcile with annual enterprise totals and 166 flow records. The analysis includes enterprises with available records; the supplied documentation does not establish a probability-based sampling design. Registry construction dates and procedures, district-level coverage, contact and response histories, reasons for the twelve exclusions, and the selection basis for the fifteen composition-sampled enterprises are not available in the supplied archive. Table S1 distinguishes these unavailable source fields from record-level completeness. Transport work is recorded flow mass multiplied by distance, and the mass-weighted mean is total transport work divided by total mass. Internal processing and downstream transfers remain separate.

Record checks examined enterprise–month keys, flow identifiers, enterprise–campaign keys and complete flow records after removing the flow identifier. No duplicate records were identified under these keys, and enterprise monthly amounts reconciled with their flow totals (Regional_Record_Checks.csv). These checks do not establish whether the same physical material was recorded again after an inter-enterprise sale. Transaction-level sender, receiver, date, lot and terminal-processing links are insufficient in the supplied archive to identify a verified non-overlapping intervention subset.

Regional allocation covers all 53 flows from the fifteen enterprises with composition records. Candidate II-PE interventions are restricted to flows recorded as washing followed by mechanical recycling into pellets. Intervention_Link_Register.csv reports the availability of terminal-processing and transaction links for these candidate flows. Verified non-overlapping coverage could not be established from the supplied evidence. Regional endpoints therefore describe conditional allocations of recorded handling flows. The other 37 enterprises contribute only to the descriptive network, with no imputed composition shares. Their observed throughput and transport characteristics are compared with those of the sampled group in Table S1.

### 2.4. Engineering Inventories and Functional Units

PE results are normalized to one tonne of pellets from the documented downstream production module. The retained batch records, as confirmed by the authors, indicate that no external additive or color masterbatch was introduced in the twelve batches; the feed was already predominantly white PE. Feed mass refers to material after cleaning and necessary drying, immediately before pelletization. Its historical cleaning route is not established by the retained records. The same downstream module is linked conditionally to alternative upstream cleaning services. Pelletization used a JSH-120 twin-screw extruder with water-ring pelletizing and cooling auxiliaries (Nanjing Chenfa Rubber and Plastic Machinery Manufacturing Co., Ltd., Nanjing, China). The pooled output-to-input ratio and electricity intensity are calculated from batch totals,

(2) $$Y=\frac{\sum_{j}P_{j}}{\sum_{j}F_{j}}\;;\;e=1000\;\frac{\sum_{j}E_{j}}{\sum_{j}P_{j}}$$

Here, *F_j_* and *P_j_* are batch feed and pellet masses in kg, and *E_j_* is batch electricity in kWh. The factor 1000 converts kg denominators to tonnes. Batch product- and feed-normalized electricity are *e*_P,*j*_ = 1000*E_j_*/*P_j_* and *e*_F,*j*_ = 1000*E_j_*/*F_j_*, respectively. The inventory uses *e*_P,pool_ = 1000∑*E_j_*/∑*P_j_* and *e*_F,pool_ = 1000∑*E_j_*/∑*F_j_*, so batches contribute according to their recorded output or feed. The authors confirmed the 1 kg reporting level for production masses; electricity entries have a 1 kWh ledger resolution. Starting and ending readings and historical calibration records are unavailable in the supplied archive; reporting resolution is therefore not an instrument error estimate. The electricity boundary includes pelletization, cutting, cooling pumps, batch preheating and standby, but excludes cleaning and drying. Production weighing used a TCS-300 platform scale (Shanghai Yousheng Weighing Apparatus Co., Ltd., Shanghai, China). PE and MIX electricity was metered with a DTSD1352 meter (Acrel Co., Ltd., Shanghai, China).

Table S7 reports all twelve batch yields, electricity intensities and descriptive statistics, using *n* − 1 for sample standard deviations. Table S8 reports leave-one-batch-out recalculation of pooled intensities and separate reporting-resolution scenarios. The latter assume either rounding to the nearest displayed unit or truncation where the historical rule is unknown. They describe consequences of stated recording rules, rather than calibrated measurement uncertainty or population confidence intervals. MIX reporting-resolution scenarios are calculated separately for MP-06; the seven different formulations are not repeated trials of that cycle.

The material account retains feed, pellets, combined solid residue and the unallocated difference between them. The residue excludes discarded metal screens; its external-incineration destination and bundled collection, loading and treatment price are enterprise-reported inputs. The unallocated difference is not assigned to moisture, volatile emissions, retained material or inventory change without supporting measurements. PE inlet moisture percentages were obtained by oven gravimetry: 60 °C for 4 h, cooling in a desiccator for 30 min, then successive 1 h drying cycles until mass changes were below 0.1% of sample mass. These conditions apply to PE pelletization feed, not the historical regional composition samples. Gravimetric loss is not a water-selective assay, and the original PE weighing pairs are unavailable in the supplied archive. Drying and analytical weighing used a GZX-9023MBE oven (Shanghai Boxun Medical Biological Instrument Corp., Shanghai, China) and an HZK-FA110S analytical balance (Huazhi (Fujian) Electronic Technology Co., Ltd., Putian, China), respectively.

Industrial cleaning electricity and dry-mass recovery are varied as explicit engineering coordinates rather than assigned a measured industrial point estimate. With *b* denoting the dry pelletization-feed requirement per tonne of pellets, the linked route requirements are

(3) $$\mu_{R}=b/r_{R};E_{R,\mathrm{clean}}=\mu_{R}e_{R,\mathrm{clean}}$$

For route *R*, *μ_R_* = *b*/*r_R_* gives tonnes of incoming dry-film equivalent per tonne of pellets, and *E_R_*_,clean_ = *μ_R_ e_R_*_,clean_ gives cleaning electricity. Here *e_R_*_,clean_ is in kWh/t cleaning feed, *r_R_* is cleaning dry-mass recovery, and *b* = 1.059396118 t dry-film equivalent/t pellets. With the assumed retained-output share *q_R_*, the corresponding coefficient is *μ_R_* = *b*/(*r_R_ q_R_*). Incoming wet mass would be *μ_R_*/(1 − *w_R_*), requiring the route-specific incoming moisture *w_R_*. The post-cleaning PE feed moisture is not applied to all regional waste. Treating recorded regional tonnes as dry-film-equivalent tonnes is an explicit scenario conversion. Industrial sand losses, regeneration and dry-cleaning water demand remain unquantified.

The wet-washing module used the industrial inventory of Martín-Lara et al. [26]. Per tonne of final low-density polyethylene (LDPE) pellets, electricity consumption was 240.0 kWh for washing/shredding, 182.4 kWh for drying, and 58.986 kWh for on-site water treatment. Other inputs and outputs were 2.0 m^3^ of tap water, 126.63 kg of deionized (DI) water for chemical dosing, 8.1 kg of polyaluminum chloride (PAC), 24 kg of 50% sodium hydroxide solution, 0.27 kg of polyacrylamide (PAM), and 2.159 m^3^ of wastewater sent off site. On-site water treatment and off-site wastewater treatment were modeled as consecutive stages. Dosing water was not added again to the wastewater volume sent off site.

The literature washing, drying and water-treatment modules are combined with the local pelletization module. The principal boundary retains all 182.4 kWh/t of wet-route drying because the documented pelletization electricity excludes drying. Subtractions of 91.2 and 182.4 kWh/t are retained only as counterfactual service-overlap tests, and do not redefine the principal service boundary. Wet-cleaning recovery values of 0.975, 0.985 and 0.992 are scenario parameters; the transferred per-tonne-pellet services remain unchanged across this axis.

For MIX, the functional unit is treatment of one tonne of the same incoming MIX-06 feed. The single MP-06 cycle processed 31.7 kg, used 9 kWh and yielded 29.4 kg product. Its remaining recorded outputs were 1.3 kg trim and 0.4 kg rejects, leaving a calculated 0.6 kg unallocated difference. The 626.2 kg incoming stock is distinct from the cycle feed. The recorded cycle electricity includes twin-screw processing, heating, melt pumping or metering, pressing and cooling auxiliaries within that cycle. Startup preheating is excluded, and additional forming or cooling services outside the recorded cycle are unquantified. Active processing lasted approximately 1–2 h. Additional electricity coordinates of 0, 20, 40 and 80 kWh/t feed are applied independently to molding (MP) and the extrusion-based comparator (EB), giving sixteen combinations. These are service-boundary scenarios rather than measured preheating requirements. The cycle used a GTE-75B twin-screw extruder (Nanjing Granuwel Machinery Co., Ltd., Nanjing, China) and a YW79-1800T hydraulic molding press (Shandong Weili Heavy Industry Machine Tool Co., Ltd., Tengzhou, China).

The EB comparator is transferred from recycled polypropylene (rPP) extrusion experiments [32]. Its reference electricity is 0.344 kWh/kg extruded product, including motor drive and barrel heating. Multiplication by a scenario product yield of 0.905 gives 311.32 kWh/t feed. The joint sensitivity uses 0.254–0.450 kWh/kg product and yields of 0.88–0.93. This conversion does not establish MIX-06 extrusion performance or a complete board-forming service. Conditioning, filtration, downstream forming, cooling and residue treatment are identified separately in Table S5.

MIX product quantities are reported separately from the common feed-treatment task. Trim and rejects were weighed and transferred free of charge to an external recycler, without on-site return or virgin-material displacement credit. The zero transaction price does not quantify their downstream environmental burden; EB non-product composition and fate also remain unknown. For PE, no common numerical acceptance specification for the dry and wet routes is available in the supplied archive. Product properties therefore cannot establish a measured qualifying fraction. Values *q* = 1.00, 0.95, 0.90 and 0.80 represent assumed retained-output shares, applied to either route in turn with the comparator at *q* = 1. Route activities, burdens, costs and feed requirements are divided by the affected *q* to supply one tonne of retained output. Non-retained pellets amount to (1 − *q*)/*q* tonnes per tonne retained; their further treatment remains an unquantified item, with no displacement credit. Nominal total-pellet capacity is multiplied by *q* once to obtain retained-output capacity.

PE melt mass-flow rate (MFR) was measured with an HT-3682V-BA melt flow tester (Guangdong Hongtuo Instrument Technology Co., Ltd., Dongguan, China) at 190 °C/2.16 kg using GB/T 3682.1-2018 [44]. Ash was determined by method A of GB/T 9345.1-2008 [45]: dried pellets were pre-charred, ignited at 600 ± 25 °C in a BZH-2.5-10TC muffle furnace (Shanghai Bozhen Instrument Equipment Manufacturing Factory, Shanghai, China) to constant mass, cooled in a desiccator and weighed. The tensile method used the test conditions of GB/T 1040.2-2022 [46], with injection-molded specimens and a speed of 50 mm/min. Specimens were prepared using a TY-7003 injection-molding machine (Jiangsu Tianyuan Testing Equipment Co., Ltd., Yangzhou, China). The retained table reports two tensile-result fields and a descriptive mean; no specimen count is inferred from these fields. EX-M1, EX-M2 and EX-M3 contain five technical specimens each, cut from MIX-01, MIX-04 and MIX-07, respectively. They are not MP-06 replicates. PE tensile and MIX tensile/flexural testing used a WDW-50 universal testing machine (Shenglin Precision Machinery Equipment (Shandong) Co., Ltd., Jinan, China).

A separate pallet test report described fork-lifting and stacking tests conducted according to GB/T 4996-2014 [47] using a JQ-8401D computer-controlled compression testing machine (Dongguan Jingqi Testing Instruments Co., Ltd., Dongguan, China), with one specimen per test. Fork-lifting tests continued to crushing failure; stacking tests continued to crushing failure or a deflection of 18.0 mm. The report characterized the load-bearing response of the tested products but had no established batch-level link to the seven MP cycles. Supporting Data S5 retains the report identifiers, test conditions and link status; the reported loads are not converted to material stress or density.

Neither the linked MIX specimen groups nor the independent pallet report establishes MP-06 product performance or equivalence to EB output. The available PE injection specimen tests likewise do not demonstrate the equal performance of finished films from the two cleaning routes. Accordingly, *q* scenarios test the consequences of assumed usable output, while the MIX comparison remains a same-feed processing task. Table 3 summarizes the process quantities and service coverage.

### 2.5. Climate Impact and Cost Calculations

Background factors were exported from SimaPro 10.1.0.4 using ecoinvent 3.10, allocation, cut-off by classification, and system-process datasets [48]. Climate characterization used the Intergovernmental Panel on Climate Change (IPCC) Sixth Assessment Report’s 100-year global warming potentials (GWP100) [49], implemented in SimaPro as “IPCC 2021 GWP100”, V1.03 [50]. Fossil, biogenic and land-transformation components are summed without normalization or weighting [51]. Table S9 and Supporting Data S6 list all eighteen characterized factors, with process names, factor identifiers, universally unique identifiers (UUIDs), reference products, units, geographic scopes and documented conversions. Database version, dataset revision, representativeness period and export date are separate metadata fields; unavailable dates are not inferred from the 2026 scenario year. The public implementation multiplies the disclosed factor snapshot by foreground activities; it does not reconstruct the proprietary background network.

East China medium-voltage electricity is the reference background, with factor 25543 equal to 0.856807240395630 kg CO_2_-eq/kWh. Reference freight factor 28198 is 0.156202592699214 kg CO_2_-eq/(t·km). Alternative electricity and freight datasets test supply and geographic proxies while retaining the database version and impact method. Conversion from a per-MJ electricity factor to a per-kWh factor uses 3.6 once. Wet-route NaOH is modeled as 12 kg active substance; a separate solvent water scenario adds the associated 12 kg water. The 126.63 kg dosing water is a distinct input. On-site water-treatment electricity and off-site wastewater treatment represent sequential services, with no second addition of dosing water to the effluent volume.

The climate impact of route r comprised quantified and unquantified contributions,

(4) $$G_{r}^{\mathrm{cov}}=\sum_{i\in \mathrm{mapped}}A_{ri}{EF}_{i}\;;\;G_{r}=G_{r}^{\mathrm{cov}}+U_{r}$$

The matched contribution sums each route activity *A_ri_* multiplied by its compatible characterized factor *EF_i_*. It is reported as covered climate impact in kg CO_2_-eq per functional unit. Unknown route-specific contributions *U* remain outside that subtotal and enter ranking-reversal calculations as a difference between routes.

The unquantified register includes mixed-solid-residue incineration, industrial sand losses and regeneration, unresolved cleaning services and comparator-specific downstream operations (Table S10). None of the eighteen exported factors matches the documented mixed PE residue; a pure-PE incineration factor is not substituted. The known treatment price supplies cost information only. A shared residue burden can cancel out in the route difference when its quantity and *q* are identical, but not automatically after unequal output scaling or in fixed-feed comparisons. The component ledger reports each matched activity × factor contribution alongside these missing services.

The quantified environmental indicator is climate change under IPCC 2021 GWP100. Human toxicity, ecotoxicity, additive migration and water scarcity were not assessed because the supplied records do not characterize relevant substances, releases or local water conditions. No newly added formulation ingredients were reported for the PE batches, and so they do not establish the absence of hazardous constituents in incoming waste. Water is reported as a physical inventory input. These results do not establish product safety or overall environmental superiority.

PE equipment investments are enterprise-reported case-study inputs. The underlying quotations and engineering records were not supplied for the independent verification of dates and service scope. Capital costs therefore remain scenario estimates, with no established equivalence between the complete dry- and wet-route equipment scopes.

(5) $$C_{\mathrm{cap}}=\frac{I-\frac{S}{{\left(1+i\right)}^{n}}}{Q}\times \frac{i{\left(1+i\right)}^{n}}{{\left(1+i\right)}^{n}-1}$$

*I* is investment, *S* is residual value, *n* is service life and *i* is the discount rate. The scenarios use *n* = 10 years, *i* = 6% and *S* = 0.05*I*. PE output capacity is 800 kg/h × 16 h/day × 300 days/year, multiplied once by utilization. At 80%, *Q* is 3072 t pellets/year; 60% and 100% give 2304 and 3840 t/year. These are capacity scenarios, not observed annual production. MIX capital uses annual feed throughput, not pellet output. Any 10,000 t/year scale case is identified separately and is not substituted for the single-line denominator.

Economic_Parameter_Sources.xlsx records the provenance and availability of case-study inputs, tariffs and analyst-selected prices. L0 comprises priced direct inputs and PE residue treatment; L1 adds labor and maintenance, and L2 adds annualized equipment capital. The PE residue charge of 400 CNY/t covers collection, loading and incineration, contributing 15.673102 CNY/t pellets without a second transport charge. The chemical-purchasing scenario uses 32.37 kg of supplied products at 2.60 CNY/kg, including 24 kg of NaOH solution, for 84.162 CNY/t pellets. Tap-water supply excludes wastewater billing. Deionized water, unmatched effluent settlement, sand losses and regeneration, and unmeasured EB forming/cooling services retain their separate unavailable-quantity or unpriced status in Table S11. All-inclusive wastewater bills are not added to overlapping electricity and chemical charges. L2 is a covered processing cost tier, not complete business cost or profit.

Extended scenarios add feed purchase and transport, separately priced auxiliary services and, where specified, product revenue. For route *R*, *p*_feed,*R*_ must use the same mass basis as *μ_R_*. *U*_cost,*R*_ retains costs whose activities or prices cannot be established. Revenue is deducted only in a named price scenario, without altering the definition of L2. The corresponding route difference is reference minus first route. MIX procurement cancels only for one tonne of the same feed purchased at the same price for both routes; PE feed coefficients generally differ. Table S11 reports procurement price scans and the net unpriced cost difference that would remove each covered margin.

$$C_{R,\mathrm{extended}}=C_{R}^{L2,\mathrm{cov}}+\mu_{R}\;p_{\mathrm{feed},R}+C_{\mathrm{transport},R}+C_{\mathrm{aux},\mathrm{priced},R}-R_{\mathrm{product},R}+U_{\mathrm{cost},R}$$

$${\Delta C}_{\mathrm{extended}}=C_{B,\mathrm{extended}}-C_{A,\mathrm{extended}}$$

### 2.6. Transport and Conditional Scenarios

Transport is calculated for each route and material segment as transported tonnes × distance × freight factor or tariff. Vehicle-loading and return assumptions represented within the selected background transport service are not applied a second time. Separate payload and empty return values are not extracted into the supplied factor snapshot, and no assumed truck capacity or round-trip multiplier is imposed. The central tariff is 0.60 CNY/(t·km), with 0.39–0.80 used as a price scan. No billing breakdown is available in the supplied archive to establish how the tariff treats return trips. Four freight datasets provide alternative transport-service proxies rather than measured loading uncertainty. PE dry-film-equivalent coefficients are not relabeled as wet-tonne requirements. Fixed transport segments enter the intercept before distance is varied.

The difference is always reference minus first route: wet minus dry for PE, and EB minus molding for MIX. A positive value favors the first route within the covered boundary. Including route-specific transport and an unknown net burden gives

(6) $$\Delta_{A}=(G_{B}^{\mathrm{cov}}-G_{A}^{\mathrm{cov}})+{EF}_{T}(\mu_{B}d_{B}-\mu_{A}d_{A})-\Delta U$$

*G*_A_^cov^ and *G*_B_^cov^ are covered process impacts; *μ* and *d* identify the transported mass coefficient and distance of each route. The unknown net burden Δ*U* = *U*_A_ − *U*_B_ is positive when the first route has a larger omitted burden. The same sign convention applies to cost comparisons of matching scope. Each transport comparison is reduced to

(7) $$\Delta (d)=A-Bd\;;d_{\mathrm{cross}}=A/B\;$$

For *B* > 0 and *A* > 0, the relevant positive crossing is *A*/*B* and the first route loses its starting advantage. For *B* < 0 and *A* < 0, the positive crossing marks a change in the opposite direction. *B* = 0 gives no distance dependence, or equality at every distance when *A* is also zero. Nonpositive roots are not converted to absolute distances. Transport outputs report the starting sign, slope and crossing status, with the positive-crossing field left empty when no relevant positive crossing exists. The supplementary results also retain signed algebraic roots, including nonpositive values.

Extra-distance slopes are the first-route transported mass coefficient multiplied by the freight factor. At a common distance the slope depends on the difference between the two mass coefficients. Thresholds outside the recorded external distance range are marked as extrapolations. An omitted process burden exhausts the covered advantage when its net difference equals the process margin; this equality is an allowance, not evidence that the omitted service can actually be supplied within it.

For MIX, A is molding and B is EB. Equal one-tonne feed, identical freight factors and shared baseline segments reduce transport to Δ*d* = *d*_MP_ − *d*_EB_. Positive Δ*d* represents a candidate molding node requiring an additional detour relative to a fixed reference node; this is a conditional rerouting test. Equal-distance transport cancels, while a negative Δ*d* favors molding on transport. No candidate plant locations or collection radii are inferred. With no additional net service burden, the reference coordinate expressions are

ΔG(Δd)=23.512140−0.156202592699214 Δd

$${\Delta C}_{L2}(\Delta d)=20.421804-0.60\;\Delta d$$

Industrial dry-cleaning electricity is treated in the same way as a variable service input. Let *G*_D,other_ contain all covered dry-route contributions except the cleaning electricity being varied, *μ*_D_ = *b*/(*r*_D_*q*_D_), and Δ*U* = *U*_D_ − *U*_W_. Then the cleaning-electricity equality is

$$G_{D}^{\mathrm{cov}}=G_{D,\mathrm{other}}+\mu_{D}\;e_{D}\;f_{\mathrm{el}}$$

$$e_{D}^{*}=\frac{G_{W}^{\mathrm{cov}}-G_{D,\mathrm{other}}-\Delta U}{\mu_{D}\;f_{\mathrm{el}}}$$

The corresponding cost equation uses a matching cost scope and the electricity tariff. Procurement or newly priced auxiliary services must enter the other-cost term before recomputing that equality. Service quantities unavailable for foreign object removal, shredding, conveying, fans or compressed air, pulse valves, dust capture, media handling and regeneration, heating/cooling, startup and downstream treatment remain in the unquantified register. Laboratory sand regeneration conditions do not supply an industrial energy-per-throughput value.

### 2.7. Scenario and Allocation Analysis

Annual quality shares are represented by ten proxies: equal-frequency weights, one monthly-throughput-weighted proxy, four single-campaign proxies and four leave-one-campaign-out proxies. The monthly proxy assigns each month to the nearest sampled month; November is split equally between October and December. Its four weight numerators are September + October + half November; half November + December + January; February + March + April; and May + June + July + August. Each is divided by the enterprise annual total. These weights use actual monthly amounts but still extrapolate sampled composition to unsampled months.

Flow mass is allocated among seven states, I-PE, I-PP, II-PE, II-PP, III, IV and U. For each flow *i* and state *s*, the allocation *x* obeys

(8) $$\sum_{s}x_{i,s}=M_{i};\sum_{i\in e}x_{i,s}=M_{e}\pi_{e,s};x_{i,s}\geq \;0\;$$

The constraints preserve each flow total and the enterprise-level state masses specified by the selected annual proxy, with nonnegative allocations. U remains in the mass account without direct dry cleaning benefit. State shares are not renormalized after excluding technically unresolved material. Leave-one-out proxies assign one-third weight to each retained campaign; a single-campaign proxy instead assigns the entire annual share to one observation.

The allocation matrices use 53 flows and seven states, giving 371 nonnegative variables and 158 equality rows, including redundant conservation equations. Candidate interventions are restricted to recorded washing-to-pellet flows allocated to II-PE. Climate and cost endpoints are obtained by separately minimizing and maximizing,

(9) $$\min/\max\;\sum_{i}\;\alpha \;{\mathrm{eligible}}_{i}x_{i,\mathrm{II}-\mathrm{PE}}(a+bd_{i})\;$$

Here, *α* is an assumed adoption fraction (0, 0.25, 0.50 or 1.00), eligible identifies the candidate flow mask, and *a* and *b* are process and common-distance coefficients. Each endpoint reports mass, transport work, output and additional feed from the same solution. Climate and cost optima are solved separately and are not claimed to occur simultaneously.

The principal fixed-feed scenario has *a* = *G*_W_^cov^/*μ*_W_ − *G*_D_^cov^/*μ*_D_ and *b* = 0 for identical external transport paths. Outputs are 1/*μ*_W_ and 1/*μ*_D_ per tonne of baseline feed, with no substitution credit for their difference. In the equal-retained-output scenario, *a* = (*G*_W_^cov^ − *G*_D_^cov^)/*μ*_W_ and *b* = (1 − *μ*_D_/*μ*_W_)*f*. Route totals and feed coefficients are first divided by their respective assumed retained-output shares. This preserves one functional denominator for activities, costs, feed supply and transport.

A single-line output-capacity constraint is applied to the modeled dry-route intervention. Equal-output scenarios additionally limit the positive extra-feed requirement, max(*μ*_D_/*μ*_W_ − 1, 0), multiplied by adopted baseline feed. The wet route is retained as the reference service and is not assigned a separate additional line-capacity constraint. Zero additional supply is compared with assumed available supply; infeasible cases remain reported as such. Multi-line and unconstrained-supply calculations are technical scenarios, not documented deployment plans.

Regional allocation uses the 252 PE electricity–recovery–qualification combinations and six utilization cases. For each process case, quality proxy and deployment constraint, the objective is regenerated and optimized. Background-factor and service boundary tests are reported separately at process level. With global capacity or supply constraints, independent verification uses the full constrained program rather than an enterprise-wise greedy allocation. Solver feasibility, conservation residuals and independently calculated coefficients are checked separately.

Uncertainty is separated into inventory structure, engineering parameters, background factors, annual quality proxies and feasible quality–flow allocations [52]. The Class II impurity upper bound is scanned at 4.5%, 5.0% and 5.5%; distances to all relevant thresholds are retained for every sample. Without measured uncertainty intervals, these are decision-rule scans, not measurement-error simulations. Scenario counts and positive-sign fractions are not experimental sample sizes or probabilities.

PE scenarios vary cleaning electricity and recovery, qualification shares in both directions, wet recovery and capacity utilization. Drying deductions are a separate boundary test. MIX scenarios vary EB yield and electricity, additional molding electricity, auxiliary inputs, costs and utilization. Industrial sand regeneration is reported through the allowed net burden together with the reported laboratory service description and the unknown industrial requirement. Finite scenario envelopes cover only the combinations explicitly calculated.

The public implementation uses Python 3.12.10, NumPy 2.4.6 and SciPy 1.18.0, with linear programs solved by scipy.optimize.linprog using HiGHS 1.12.0. The single entry point reproduce_all.py regenerates process contributions, crossing conditions and regional endpoints from the disclosed comma-separated values (CSV) inputs and characterized-factor snapshot. Workbook inputs are matched by record identifier; result tables and figure sources are checked against generated outputs. revision_checks.py adds batch statistics, reporting-resolution, procurement and record-consistency checks. The final archive documents execution commands, actual runtime versions, input hashes, numerical checks and output-to-table/figure mappings. Computational consistency does not independently validate source measurements or the proprietary background database.

## 3. Results

### 3.1. Regional Material Flows and Temporal Changes in Feedstock Quality

**Figure 2:**
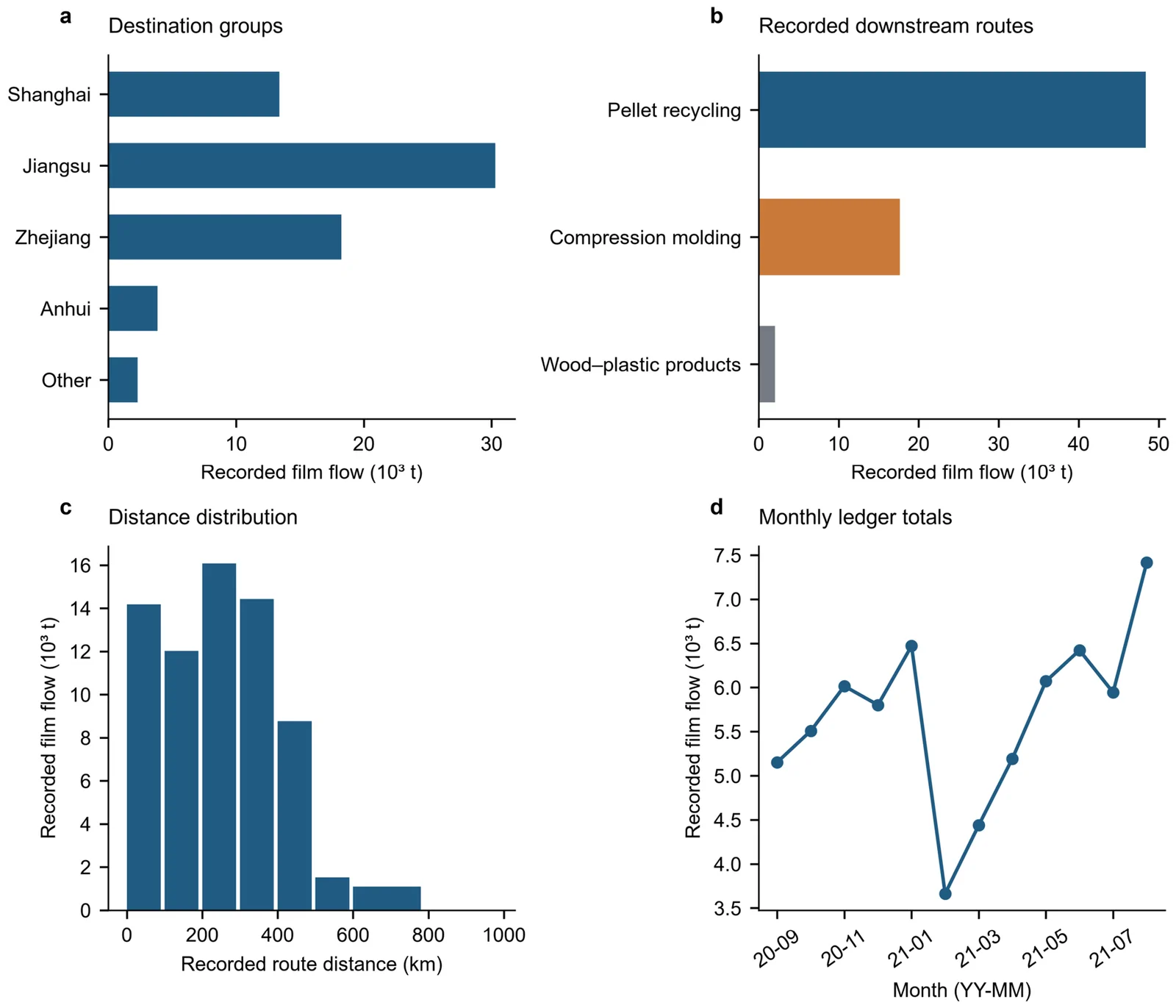
Recorded regional film-handling network. (**a**) Destination groups, with internal processing included in the Shanghai group; (**b**) downstream routes; (**c**) distribution of recorded flow mass across distance bins; and (**d**) monthly totals from 52 enterprises. The 166 flows comprise 5870 t internal handling and 62,226 t downstream transfer, totaling 68,096 t. The 624 enterprise–month records span September 2020–August 2021. These quantities may include repeated handling after resale and are not deduplicated waste generation.

The 52 enterprises recorded 68,096 t of film handling, comprising 5870 t processed internally and 62,226 t transferred downstream. The contextual total for all recorded plastic recycling was 315,260 t; it is not the film denominator used in allocation (Figure 2). Film transport amounted to 16.826 million t·km, with a mass-weighted mean distance of 247.10 km using 0.1 km recorded distances. The fifteen enterprises with composition records accounted for 53 flows and 21,617.997 t, or 31.75% of recorded film handling. The other 37 enterprises accounted for 113 flows and 46,478.003 t. Median enterprise annual handling was 1173.808 versus 1094.713 t, and mass-weighted distances were 245.108 versus 248.025 km, respectively (Table S1). These descriptive similarities do not remove possible selection bias or establish coverage of unique regional waste.

**Figure 3:**
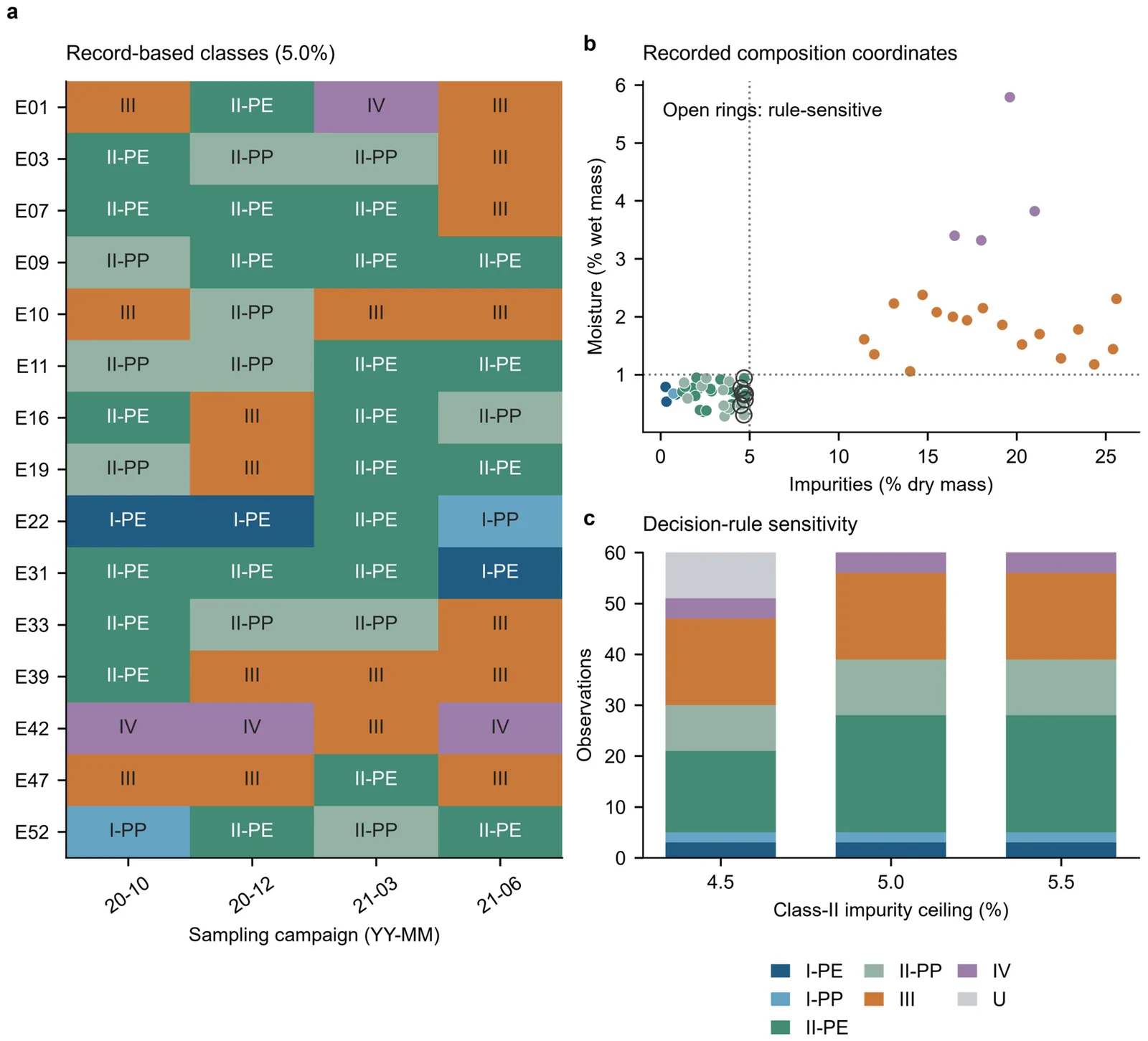
Record-based quality observations and rule sensitivity. (**a**) Four observations from each of the 15 enterprises at the 5.0% Class-II impurity ceiling, (**b**) recorded impurity and moisture coordinates and (**c**) class counts after changing that ceiling. Open rings identify observations whose recorded class changes across the three impurity ceilings. U remains in every total. Dotted references mark 5.0% impurities and 1.0% moisture, not measurement error limits. Technical applicability is unconfirmed and recorded separately as U; it is not represented by the record-based colors.

Feedstock quality varied both between enterprises and between sampling periods within enterprises. Under the baseline Class II impurity upper bound of 5.0%, all 60 samples were assigned uniquely to a quality class: 5 to Class I, 34 to Class II, 17 to Class III, and 4 to Class IV. Class II comprised 23 PE and 11 PP samples (Figure 3). Of the 15 enterprises, 13 changed class across the four sampling periods, so a fixed enterprise-level classification did not capture the observed variation across campaigns.

### 3.2. Process Outputs, Energy Demand, and Route Inventories

**Figure 4:**
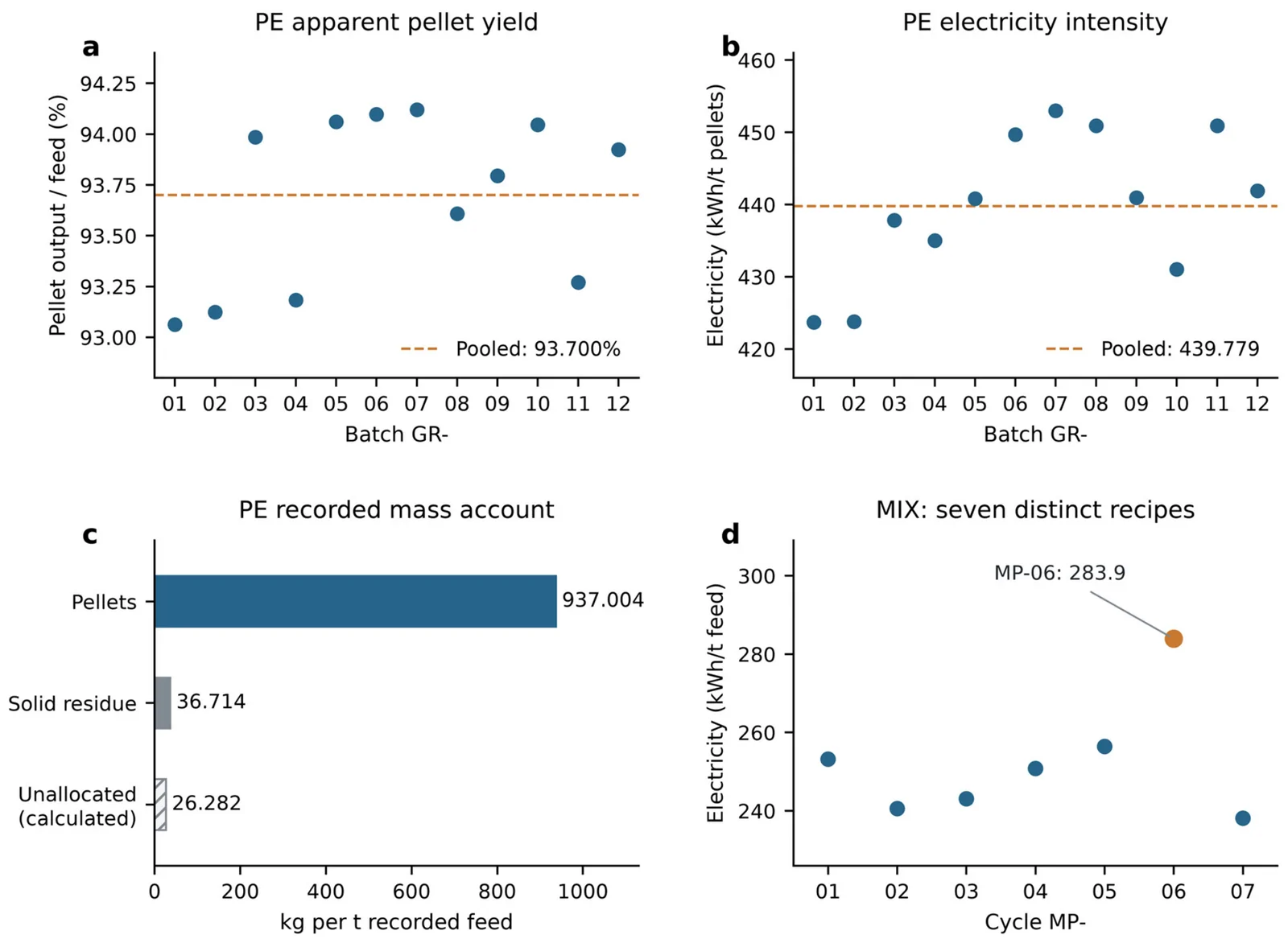
Batch-level records and PE mass account. (**a**) Apparent pellet yield and (**b**) electricity per tonne of pellets for all twelve PE batches, with batch identifiers and pooled reference lines. (**c**) PE outputs per tonne of recorded feed: 937.004 kg pellets, 36.714 kg recorded solid residue and 26.282 kg calculated unallocated difference. Distinct fill marks the difference term. The 299.524 kg inlet gravimetric loss calculation is a diagnostic only and is not plotted as an independently measured output. (**d**) Seven MIX recipe-specific cycles, with MP-06 highlighted. PE electricity includes pelletization auxiliaries, preheating and standby, but excludes cleaning/drying; MIX processing excludes startup preheating. No instrument error bars are inferred from reporting resolution, and the MIX formulations are not repeated trials of MP-06.

The twelve PE batch records total 40,796 kg feed, 38,226 kg pellets, 16,811 kWh and 9.95 m^3^ make-up water. Ratios of totals give 93.700% apparent pellet yield, 439.779 kWh/t pellets and 412.075 kWh/t recorded feed. Across individual batches, yield averaged 93.690% with a sample standard deviation (SD) of 0.418 percentage points and range 93.062–94.119%. Product-normalized electricity averaged 439.961 kWh/t pellets (SD 10.199; range 423.717–452.983), while feed-normalized electricity averaged 412.217 kWh/t feed (SD 10.557; range 394.321–426.345). The pooled and arithmetic means differ because batch masses differ. Figure 4 and Table S7 report all batch values. Make-up water was 0.2603 m^3^/t pellets, and the recorded inlet moisture fractions give b = 1.0594 t dry-film equivalent/t pellets.

The PE mass account is 40,796 kg feed = 38,226 kg pellets + 1497.8 kg recorded solid residue + 1072.2 kg unallocated difference. These terms represent 93.700%, 3.671% and 2.628% of feed, respectively; the combined non-pellet fraction is 6.300%. Calculated inlet gravimetric loss totals 299.524 kg. Provisionally assigning all of it to the difference would still leave 772.676 kg unresolved, without identifying actual moisture, volatile, retained-material or other losses. Figure 4 therefore shows pellets, recorded solid residue and unallocated difference as three separate terms. The residue’s reported incineration destination and treatment price do not establish its missing climate factor.

Leaving out one PE batch at a time changed the pooled pellet electricity intensity to 438.572–441.430 kWh/t. Assuming nearest-unit rounding of integer-kWh and integer-kg entries gave an envelope of 439.553–440.005 kWh/t pellets (Table S8). For MP-06, rounding 9 kWh and 31.7 kg to the reported resolutions gave 267.717–300.158 kWh/t feed. At the other reference settings, these MP-06 scenarios moved the climate distance crossing to 61.4–239.4 km and the L2 crossing to 11.8–56.2 km. Truncation scenarios are reported separately, retaining nonpositive-root statuses. These ranges characterize sensitivity to the stated recording rules, not measured instrument uncertainty.

The contrast between the PE and MP-06 recording rule envelopes reflects the scale of the underlying records. The PE intensity aggregates twelve batches and 16,811 kWh, whereas MP-06 is based on a single 9 kWh cycle. The assumed integer-kWh recording rule therefore has a larger proportional effect on the latter calculation. The MP-06-derived transport crossings should consequently be interpreted together with the reported recording rule sensitivity, rather than as exact operational limits.

The twelve PE pellet batches had MFR values of 1.11–1.71 g/10 min, ash contents of 0.11–0.66% and reported tensile means of 19.02–22.76 MPa. These ranges describe the recorded products and are not acceptance thresholds. No external additive or color masterbatch was reported in the batches. Customer use was reported at 30 wt% recyclate in virgin–recycled resin blends for heavy-duty packaging films. Numerical acceptance limits are unavailable in the supplied archive, and the injection-specimen tensile results do not establish final-film properties. Accordingly, the *q* scenarios do not estimate observed acceptance or rejection rates.

Seven recipe-specific molding cycles processed 213.7 kg and produced 197.2 kg using 54 kWh. Their pooled output ratio is 92.3% and electricity intensity is 252.7 kWh/t feed; these totals do not estimate replication uncertainty for MIX-06. The MP-06 record gives 927.4 kg product and 283.9 kWh per tonne of feed. It also gives 41.0 kg trim, 12.6 kg rejects and a calculated 18.9 kg unallocated difference per tonne. At the EB yield coordinate of 0.905, non-product mass is 95.0 kg/t feed with unresolved composition and fate.

Five specimens were tested for each of MIX-01, MIX-04, and MIX-07. Group-mean tensile strengths ranged from 18.55 to 20.74 MPa, and flexural strengths from 23.87 to 27.33 MPa (Table S12 and Figure S1). An independent pallet report tested one item in each of the fork-lifting and stacking tests, with terminal loads of 1012 and 4998 kgf, respectively. The relationship of these tests to the material sample groups and MP cycles is described in Section 2.4.

### 3.3. Conditional Climate and Covered-Cost Differences

**Figure 5:**
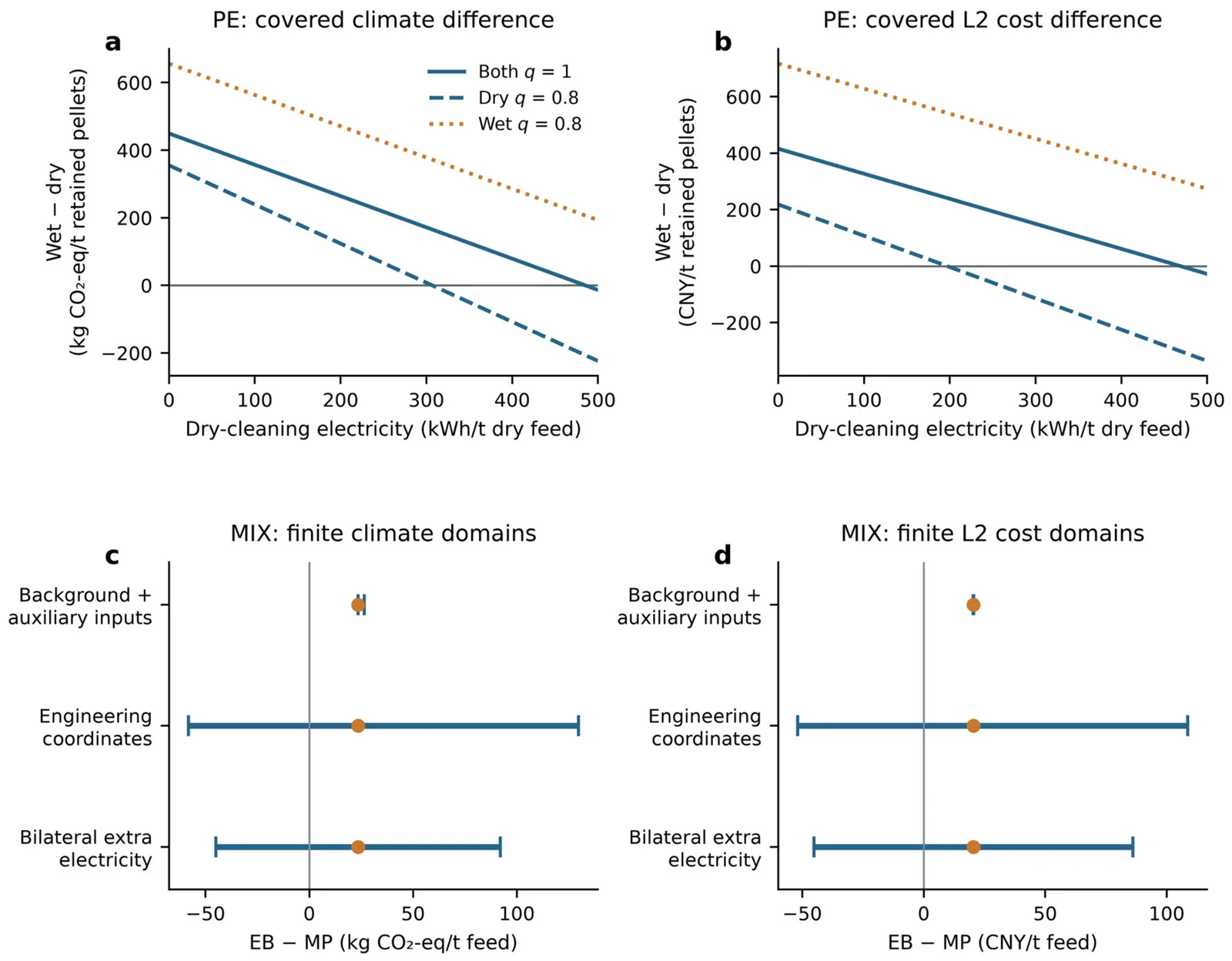
Conditional differences in covered climate impact and cost. (**a**,**b**) PE dry-electricity coordinates at *r*_D_ = 0.98 and *r*_W_ = 0.985, with one-sided *q* stress tests; *q* is not a measured product acceptance rate. (**c**,**d**) MIX intervals across the disclosed background/auxiliary, engineering and additional-service coordinates; orange points show the reference coordinate. MP denotes molding. Positive differences favor the first route within the covered subtotal. The intervals are finite parameter domains, not confidence intervals or demonstrated industrial operating ranges; unknown net burdens can shift the boundaries.

For *r*_D_ = 0.98, *r*_W_ = 0.985 and *q*_D_ = *q*_W_ = 1, the covered PE climate ranking changes at a dry cleaning electricity coordinate of 485.2 kWh/t dry cleaning feed with East China medium-voltage electricity. The corresponding L2 cost coordinate is 468.8 kWh/t (Figure 5). These are conditional equality values, not estimates of actual industrial demand. They assume the wet service retains its full drying load and all other unresolved net burdens remain outside the covered subtotal.

Two coordinates illustrate the crossing. At *e*_D_ = 100 kWh/t dry feed, covered PE impacts are 469.8 and 826.5 kg CO_2_-eq/t pellets, and L2 costs are CNY 878.0 and 1204.9/t for dry and wet routes. Raising *e*_D_ to 500 kWh/t gives reference-minus-dry differences of −13.7 kg CO_2_-eq/t and −27.7 CNY/t. Assumed retained-output shares also change the per-tonne denominator in both directions. The 252 electricity–recovery–retained output combinations span −242.0 to 656.0 kg CO_2_-eq/t; this span describes the selected mathematical coordinates, not an observed operating distribution.

At the MIX reference coordinate, covered molding and EB climate impacts were 243.3 and 266.9 kg CO_2_-eq/t feed, and L2 costs were 972.8 and 993.2 CNY/t feed. EB-minus-molding margins were therefore 23.5 kg CO_2_-eq/t and 20.4 CNY/t. In the disclosed component ledger, the difference between 311.32 and 283.91 kWh/t feed accounts for 23.484 kg CO_2_-eq/t, while the net contribution from the assumed water and wastewater inputs is 0.028 kg CO_2_-eq/t. The reference covered climate margin thus arises almost entirely from the electricity difference within the specified service boundary.

The L2 margin reflects a different balance. Relative to EB, the molding scenario has lower electricity, water, labor and maintenance costs by 22.475, 0.066, 25.000 and 30.000 CNY/t feed, respectively, but a higher annualized capital cost by 57.119 CNY/t feed. The capital term offsets much of the other covered cost differences, leaving a net margin of 20.422 CNY/t feed before unequal transport and unpriced services. These are component differences at the stated cost coordinates, rather than observed profit differences.

The 64 background/auxiliary-input combinations retained positive climate differences of 23.5–26.4 kg CO_2_-eq/t. Across 5184 engineering combinations, climate differences ranged from −58.2 to 129.7 kg CO_2_-eq/t and L2 differences from −51.9 to 108.6 CNY/t. The sixteen bilateral additional-electricity combinations spanned −45.0 to 92.1 kg CO_2_-eq/t and −45.2 to 86.0 CNY/t. Sign stability therefore applies only to the tested background and auxiliary-input domain. The engineering and additional-service scenarios change the electricity requirements or the assumed EB yield and can reverse the ordering. These finite scenario sets do not establish the probability of either route being preferable.

Procurement affected PE differences because the reference feed coefficients were *μ*_D_ = 1.081016447 and *μ*_W_ = 1.075529054 t dry-film equivalent/t pellets. For wet-route purchase price *p*_W_ and dry-route premium δp on that same basis, the extended cost difference was 326.920868 + (1.075529054 − 1.081016447)*p*_W_ − 1.081016447δp − Δ*U*_cost_ at the low-electricity coordinate, before any unequal transport or separately specified revenue. Table S11 scans *p*_W_ = 0, 500, 1000, 2000, 4000 and 6000 CNY/t with δp = −500, −250, 0, 250 and 500 CNY/t, excluding negative purchase prices. These are assumed coordinates, not market observations. For MIX, common procurement canceled only under equal feed quantity and price. A net unpriced molding cost of 20.421804 CNY/t feed would exhaust its reference L2 margin even at equal transport distance. Separate product price scenarios retain revenue as a distinct term; freely transferred trim and rejects receive no molded-product revenue.

The documented PE downstream module used 0.2603 m^3^/t pellets of make-up water. Adding wet-route services gave 2.2603 m^3^ ordinary water plus 126.63 kg deionized dosing water, equivalent to 0.12663 m^3^. The covered freshwater input was therefore approximately 2.3869 m^3^/t pellets, with 2.159 m^3^/t separately reported as wastewater sent off site. Industrial dry-cleaning water demand is not established, so 0.2603 m^3^/t is not a total dry-route water balance and does not support a whole-route water-saving percentage. MIX water inputs of 0.060 and 0.080 m^3^/t feed remain auxiliary-input scenarios. These quantities are physical inventory flows, not water-consumption or scarcity-footprint results.

### 3.4. Break-Even Conditions for Transport and Additional Climate Burdens

**Figure 6:**
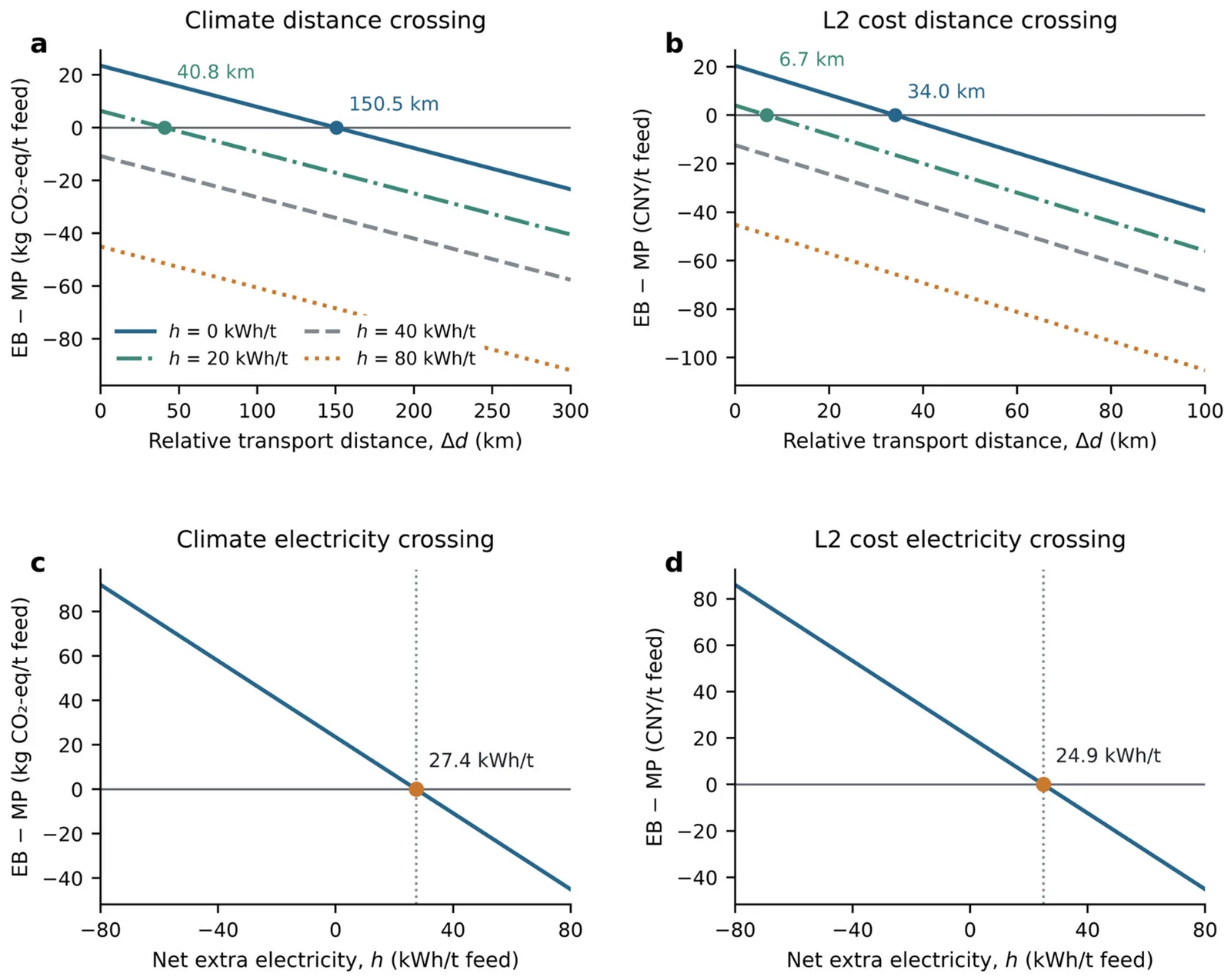
MIX distance and electricity conditions at the reference coordinate. (**a**,**b**) Climate and L2 differences versus relative distance Δ*d* = *d*_MP_ − *d*_EB_ for net additional molding electricity *h* = 0, 20, 40 and 80 kWh/t feed. At *h* = 0, crossings occur at 150.5 and 34.0 km. At *h* = 20, they are 40.8 and 6.7 km; the *h* = 40 and 80 cases are unfavorable at the origin for both indicators. (**c**,**d**) Differences versus *h* = MP_extra_ − EB_extra_, crossing zero at 27.4 and 24.9 kWh/t feed. The calculation uses *f*_el_ = 0.856807240396 kg CO_2_-eq/kWh, *f*_T_ = 0.156202592699214 kg CO_2_-eq/(t·km), electricity price 0.82 CNY/kWh and freight price 0.60 CNY/(t·km). Zero *h* is a difference coordinate, not evidence that omitted services are absent. Crossings identify conditional equality points and do not locate collection radii.

At the MIX reference coordinate, the additional relative distances Δ*d* = *d*_MP_ − *d*_EB_ that removed the covered L2 and climate margins were 34.0 and 150.5 km, respectively (Table 4; Figure 6). Between these values, the covered climate difference remained positive while the L2 difference was negative. Equal distances canceled for the same one-tonne feed, irrespective of the shared baseline distance. With net additional molding electricity *h*, the climate intercept became 23.512140−0.856807240396 *h* and the cost intercept 20.421804−0.82 *h*. At *h* = 20 kWh/t feed, the corresponding distance crossings fell to 40.8 and 6.7 km. At *h* = 40 or 80, both reference margins were already negative at Δ*d* = 0, with no positive loss-of-advantage crossing.

PE transport slopes depend on recovery and qualification through the feed coefficients. At *e*_D_ = 100, *r*_D_ = 0.98, *r*_W_ = 0.985 and *q*_D_ = *q*_W_ = 1, these coefficients are 1.0810 and 1.0755 t dry-film equivalent/t pellets. Additional dry-route transport consumes the covered process margin, whereas a common distance adds only the difference in feed mass. The extra-distance climate crossing is 2113.0 km, beyond the recorded network maximum of 761.1 km; the L2 crossing is 504.0 km, within that network range. Neither range comparison validates a specific PE route. At *e*_D_ = 500, the dry route already has a higher covered process burden, so a positive loss-of-advantage distance does not exist.

The reference MIX climate margin was exhausted by 27.4 kWh/t feed of net additional molding electricity; the corresponding L2 threshold was 24.9 kWh/t feed. Additional EB services enter with the opposite sign. For PE, an unquantified net dry-route burden shifts the cleaning electricity equality downward according to Section 2.6. The allowable burden is reported beside the unknown industrial requirement for sand loss and regeneration. The laboratory records include ten entries labeled as reuse tests and three labeled as post-heating tests; the reported heating conditions were 600 °C for 5 h. These records provide no measured industrial energy per unit throughput.

### 3.5. Regional Scenarios with Incomplete Quality–Destination Matching

At the 5.0% rule, the equal-frequency proxy assigns 8761.8 t to II-PE. Feasible allocation to recorded washing-to-pellet flows is 3490.9–7272.6 t before adoption or capacity restrictions. The monthly-weighted proxy changes the candidate interval to 2728.7–6605.2 t. These quantities retain the recorded-flow basis and are not a verified, nonduplicated volume of technically suitable material.

**Figure 7:**
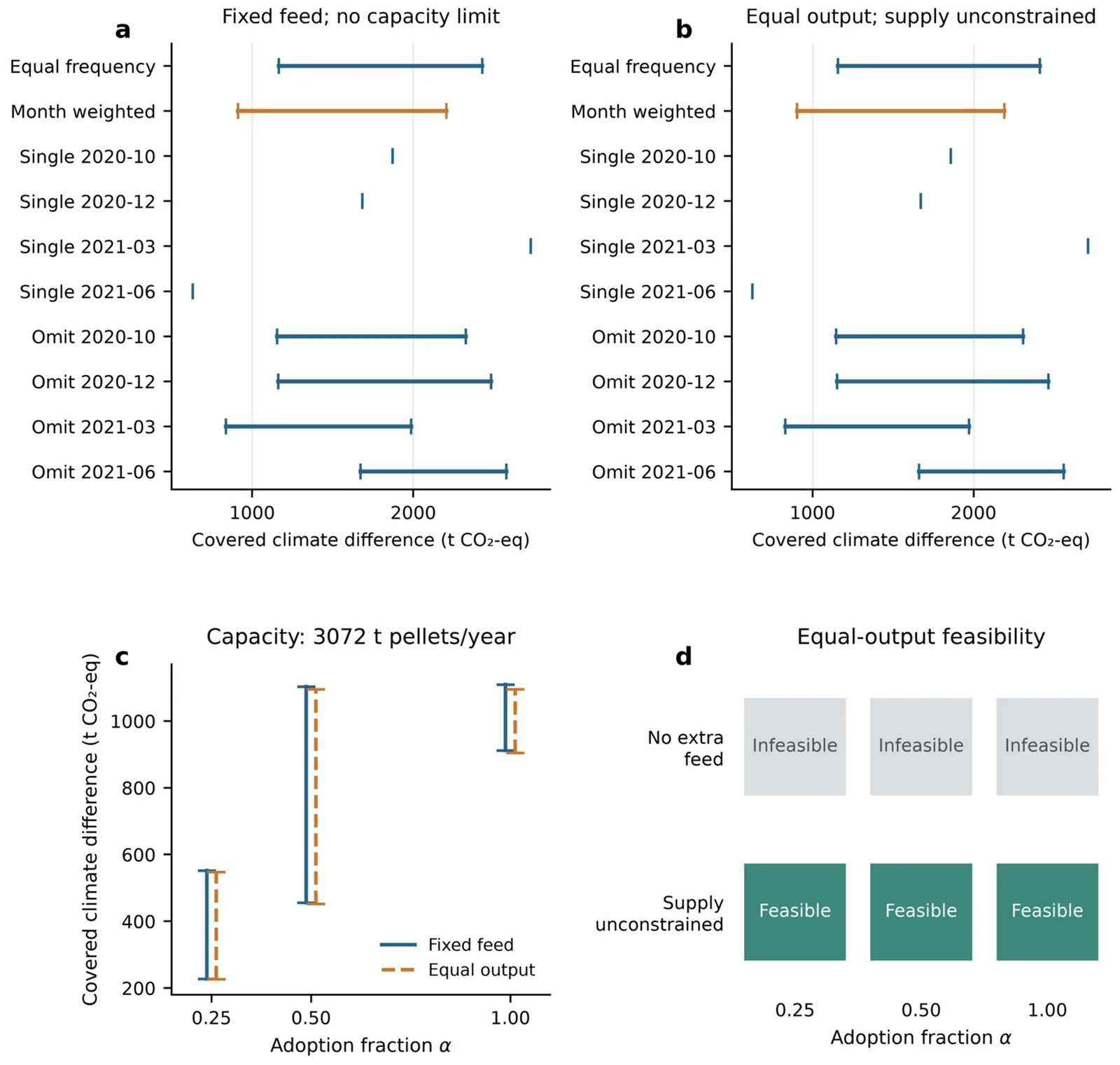
Conditional regional allocation results for the sampled enterprises. (**a**,**b**) Climate endpoints across ten annual quality proxies at the 5.0% impurity ceiling and full adoption, without a capacity ceiling; equal-output cases allow additional feed. (**c**) Endpoints with a nominal dry-route intervention capacity of 3072 t pellets/year and the monthly proxy. (**d**) Equal-output feasibility under the same dry-route capacity, contrasting zero additional feed with unconstrained supply. All panels use the hypothetical PE coordinates *e*_D_ = 100 kWh/t dry feed, *r*_D_ = 0.98, *r*_W_ = 0.985 and *q*_D_ = *q*_W_ = 1. Intervals are separately optimized allocations of recorded, non-deduplicated flows; the flow tonnage is conditionally treated as dry-film equivalent, and no verified physical saving is claimed.

At the illustrative *e*_D_ = 100, *r*_D_ = 0.98, *r*_W_ = 0.985 and *q*_D_ = *q*_W_ = 1 setting, the fixed-feed, full-adoption scenario gives covered climate differences of 1165.8–2428.7 t CO_2_-eq under the equal-frequency proxy. Dry-route output is 16.5–34.3 t lower. Corresponding cost endpoints are CNY 1.076–2.241 million. The monthly weighted climate endpoints are 911.2–2205.8 t CO_2_-eq (Figure 7a). These calculations conditionally treat recorded tonnes as dry-film-equivalent feed and exclude unmatched net burdens.

Reducing the Class II impurity upper bound to 4.5% moved seven II-PE and two II-PP observations into classification U, changing candidate masses and regional endpoints. Raising it to 5.5% left the 5.0% assignments unchanged. Table S6 summarizes these decision-rule sensitivities; regional_inputs/threshold_distances.csv reports the sample-level distances to moisture, polymer, paper and equipment thresholds. Technical applicability has not been verified, and the recorded precision does not establish a measurement-error distribution.

Holding retained pellet output constant at the same coordinate gave covered climate endpoints of 1157.3–2411.0 t CO_2_-eq and required 17.8–37.1 t additional dry-feed equivalent. Zero available extra supply made these prescribed full-adoption cases infeasible. A single line producing 3072 t total pellets/year also restricted eligible full-adoption allocations. The constraint was imposed within optimization, rather than applied afterward to an unconstrained endpoint.

Lower adoption fractions reduce converted quantities, but feasibility still depends on quality allocation and capacity. When *q*_D_ is below one, nominal total-pellet capacity is multiplied by *q*_D_ to obtain retained-output capacity. Cases unable to satisfy prescribed adoption, output and feed-supply conditions retain an infeasible status. They are not recoded as zero-intervention outcomes.

Wet recovery values of 0.975, 0.985 and 0.992 change the direction and magnitude of equal-output feed adjustments (Figure S2). Changes in assumed retained-output shares can exceed the recovery effect. Each combination is solved with its own feed coefficients; product-output, feed-supply and transport quantities at an endpoint come from one feasible allocation.

The high-electricity dry coordinate reversed regional covered climate and cost rankings under otherwise unchanged conditions. Each endpoint file separates variation within a fixed process-and-proxy allocation problem from changes in annual proxy, process inputs or deployment constraints. The regional analysis uses PE coordinates; MIX background and engineering sensitivities remain separate process comparisons in Section 3.3.

## 4. Discussion

### 4.1. Feedstock Quality and the Basis for Route Comparison

Thirteen of fifteen enterprises changed record-based class across the four campaigns. An enterprise label therefore cannot identify the quality of each dispatch throughout the year, and four observations do not measure a complete annual quality distribution. Previous flexible-film studies link composition and process improvements to recycling performance and product value [7,8]. More recent experiments connect sorting quality with residual inclusions and mechanical performance, while also recognizing the effects of washing and melt filtration [17]. This explains why a feedstock class can guide candidate-route screening without certifying the suitability of the resulting product. In the present records, an assigned zero does not establish component absence, and class membership must remain separate from technical applicability.

A common output mass does not establish equivalent product service. In the PE scenarios, a lower assumed retained output share increases the feed and process requirements per retained tonne while reducing the retained output capacity of a line. The parameter *q* therefore expresses the consequences of usable-output assumptions; it is not an acceptance rate estimated from the available property tests. The historical PE feed was already cleaned and dried, so its production record cannot identify the upstream cleaning route. For MIX, fixing incoming feed preserves the treatment comparison while retaining different product quantities and functions. This distinction is consistent with the separate roles of treatment, product function and substitution in recycling assessment [10,19,21]. Ekvall et al. [33] account for different end-use services through a common basket of functions. Here, unverified final-product equivalence limits the MIX comparison to the specified feed-treatment service, without product-substitution credits.

### 4.2. Process Differences Under Changing Transport and Cost Conditions

The PE comparison separates the documented pelletization module from the upstream cleaning services. At the same output quantity and *q*, the common downstream contribution cancels from the route difference. The comparison is then governed by the cleaning services, their recoveries and any omitted net burdens. The recorded 439.8 kWh/t pellets does not establish industrial dry-cleaning demand. The climate equality of 485.2 kWh/t dry cleaning feed and the L2 equality of 468.8 kWh/t are screening conditions at the stated settings. They are not industrial operating values validated by the production records. The wet benchmark supplies washing, drying and water treatment services [26]. Because the production meter excludes cleaning and drying, the principal boundary retains the full wet drying load. Subtracting it tests a different service assumption. Actual preparation, auxiliaries and media regeneration must be accounted for before applying either equality to a plant.

For MIX, the small reference margins leave limited room for unequal services. The covered climate difference is exhausted by 27.4 kWh/t feed of net additional molding electricity, and the L2 difference by 24.9 kWh/t at the reference tariff. Additional EB services enter with the opposite sign. The EB parameter comes from recycled-PP extrusion on a small extruder [32], rather than a complete MIX-06 board-making line. Conditioning, forming, cooling and startup allocation therefore need comparable coverage before interpreting a plant-level difference. This scale and service distinction is consistent with recent film-recycling LCA comparing laboratory and industrial processing [34]; that study does not supply an industrial electricity value for the present feed.

The different transport crossings explain why a climate advantage can coexist with a covered cost disadvantage. At the reference service and price coordinates, an additional relative molding distance below 34.0 km retains both covered advantages. Between 34.0 and 150.5 km, the climate difference remains positive but the L2 difference is negative; equality occurs at the respective crossing. Adding 20 kWh/t feed of net molding electricity reduces the L2 and climate crossings to 6.7 and 40.8 km, respectively. Service completion and transport therefore act jointly on the available margin. These distances describe a detour relative to the EB route with matched baseline transport, not a collection radius or the total length of either route.

The cost decomposition in Section 3.3 shows that the higher molding capital charge offsets much of its lower electricity, water, labor and maintenance costs. A net unpriced molding cost of 20.4 CNY/t feed would remove the remaining reference L2 margin even at equal transport distance. PE procurement behaves differently because its feed coefficients depend on recovery and assumed retained output; common feed prices do not generally cancel. They cancel in the MIX difference only for equal quantities of the same feed purchased at the same price. Utilization and product prices can further change economic outcomes, as reflected in techno-economic studies [29,30,53]. Recent flexible-packaging work also evaluates environmental impacts and life cycle costs while retaining material losses and limits on recyclate uptake [35]. The present L2 comparison remains a covered processing cost result; its procurement scans are assumed coordinates, and product revenue and unpriced services must be considered separately before assessing business viability.

### 4.3. Regional Inference with Incomplete Quality–Flow Matching

Annual proxy choice and within-enterprise flow allocation address different unknowns. Moving from equal-frequency to monthly-throughput weighting changes the candidate recorded flow interval from 3490.9–7272.6 to 2728.7–6605.2 t. The rule used to extend four observations across the year therefore changes how much material can enter the candidate intervention before capacity is considered. Within each proxy, the remaining interval reflects alternative quality–destination assignments that preserve the recorded flow totals and the imposed enterprise quality shares. It is not a confidence interval for measured regional savings. Single-campaign proxies can collapse the interval by assigning each enterprise one quality state, but add no flow-specific composition evidence. Separating the proxy and allocation results identifies which part of the range arises from annual weighting and which arises from missing quality–destination links [41].

The 53 sampled enterprise flows represent 31.75% of recorded film handling mass, not a verified fraction of independently generated regional waste. Without transaction links and confirmed intervention sites, physical savings cannot be deduplicated. Capacity and additional feed limits place further restrictions on record-based scenarios. A single-line constraint can make a prescribed adoption fraction infeasible, and an equal-output comparison can require feedstock not documented as available. These outcomes concern the imposed deployment assumptions, not the existence of a feasible unrestricted recycling network.

At the illustrative settings of *r*_D_ = 0.98, *r*_W_ = 0.985 and *q*_D_ = *q*_W_ = 1, the dry route needs slightly more incoming feed per retained tonne because its assumed cleaning recovery is lower. Holding feed fixed therefore lowers dry-route pellet output; holding retained output fixed instead creates an additional supply requirement. These comparisons concern different services, and their climate differences are not interchangeable savings estimates. Capacity then limits the prescribed intervention, while a lower *q* reduces retained-output capacity. A favorable process-level difference can consequently coexist with an infeasible regional intervention at the specified adoption fraction. This identifies a failed deployment condition; it does not imply that every possible recycling configuration is infeasible.

### 4.4. Product Function and Transferability

The documented PE products were reportedly used in films containing 30 wt% recyclate, but numerical acceptance limits common to the cleaning routes are unavailable in the supplied archive. MFR, ash and injection-specimen tensile results describe those pellets within their test conditions. Polymer composition, residual contaminants, filtration and thermal history can affect product properties. We therefore vary *q* as an assumed retained-output share; the reported property ranges do not define acceptance criteria or measured rejection rates.

The specimen and pallet evidence has the scope described in Section 2.4 and does not establish MP-06 equivalence to EB output. Transferring recycled-PP extrusion electricity [32] is useful for defining comparator conditions, but conditioning and downstream forming may change both energy demand and output properties. Rated loads, service life and functional substitution require evidence for the product and formulation actually compared.

Displacement by recycled products depends on technical functionality [19,20] and market conditions [21], while recycling quality also involves the time materials remain in use [10]. The main analysis excludes credits for virgin material displacement and applies to cases with similar feedstocks and explicit process and output requirements. Transfer to another setting requires updating these conditions together with the electricity and transport parameters.

### 4.5. Limitations

The assessment combines records collected at different times for different purposes. The 2020–2021 ledgers and composition observations characterize the recorded handling network, whereas later production records describe separate process modules. Combining them assumes the transferability of the specified services. The supplied archive does not establish probability-based enterprise selection, and four composition observations per enterprise cannot measure a full annual quality distribution. Similar observed throughput and transport characteristics between sampled and unsampled enterprises do not remove selection bias.

Composition data are compiled records with incomplete sample-level weighing and identification evidence in the supplied archive. Oil-associated material lacks a validated oil-specific assay, and other impurities are calculated by difference. These records support operational classification but do not establish industrial route suitability. Transaction links are also insufficient to exclude repeated recording after resale. Regional endpoints therefore remain conditional allocations of handling records, with verified non-overlapping intervention coverage unavailable.

The twelve PE batches describe one production module and period. Batch dispersion, leave-one-batch-out checks and recording-rule scenarios do not establish instrument accuracy or variability across plants. The unresolved PE mass difference has not been assigned to measured emissions. MP-06 contributes one processing cycle and excludes startup preheating; the transferred rPP extrusion parameter does not establish a complete MIX-06 board-making service. Unquantified services on either side can change the thresholds.

Retained-output shares are assumptions rather than measured acceptance rates, and available tests do not establish equivalent final-product service. Environmental results cover climate change and physical water inputs, leaving toxicity, additive migration, product safety and water scarcity unassessed. Procurement scans and explicit unpriced services extend the economic comparison but do not make it a complete business-cost or profit assessment. The findings apply to the specified process, quality, transport, capacity and feed-supply conditions.

## 5. Conclusions

The assessment connects incomplete enterprise-level quality observations to recorded material flows while preserving the distinction between fixed-feed and equal-retained-output tasks. The network contains 68,096 t of film handling across 52 enterprises; four observations at each of fifteen enterprises show changing record-based classes. Separately documented PE production records give a pooled pelletization intensity of 439.8 kWh/t pellets. The network, composition and production records were collected in different periods and cannot be treated as a single contemporaneous dataset.

Dry-cleaning electricity thresholds identify conditional equality points rather than measured industrial requirements. For the same MIX-06 feed, the processing record and transferred extrusion reference give covered climate and L2 cost differences of 23.5 kg CO_2_-eq/t and 20.4 CNY/t, respectively. Additional relative molding transport removes those margins at 150.5 and 34.0 km. Engineering variation, procurement conditions and unquantified services can change the corresponding route ranking. The different climate and cost crossings show that a favorable climate comparison does not necessarily imply a covered cost advantage at the same relative transport distance.

Regional allocation ranges depend on both annual quality weighting and the assignment of enterprise-level quality shares to recorded flows. Capacity and extra-feed constraints can make prescribed interventions infeasible, while fixed-feed scenarios retain their output differences explicitly. Confirmed technical applicability and transaction-level intervention links are still needed to translate these record-based scenarios into non-overlapping physical interventions. The regional scenarios identify the process, allocation and supply conditions that must hold together before a favorable process margin can be translated into a feasible intervention.

## Figures and Tables

**Table 1:** Data sources, observational units, and analytical uses. PE, polyethylene; MIX, mixed feed; EB, extrusion-based engineering reference.

Data Type	Date, Observational Unit and Evidence Type	Analytical Role; Combination Assumption; Inference Boundary
Regional ledgers	September 2020–August 2021; 52 enterprises × 12 months; 166 flows. Evidence: enterprise-derived record.	Role: recorded handling, destinations and distances. Combination: preserve ledger totals when linking later process modules. Boundary: possible repeat handling after resale; not unique regional generation or historical process performance.
Composition	October and December 2020; March and June 2021. Fifteen enterprises × four campaigns = 60 compiled records; reported identification/separation procedure.	Role: record-based classes and annual quality proxies. Combination: stated temporal weighting and admissible allocation to enterprise flows. Boundary: no imputation to the other 37 enterprises; technical applicability and sample-level analytical confirmation unresolved.
Legacy cleaning	Enterprise-derived descriptions of laboratory cleaning.	Role: source associations and laboratory service description. Combination: common material origins only. Boundary: no continuous cleaning–pelletization experiment or industrial electricity/media inventory.
PE production	2–13 November 2024; 12 batches; 40,796 kg rounded feed. Evidence: enterprise-derived record.	Role: pelletization mass, electricity and product properties. Combination: conditional downstream module for dry and wet cleaning. Boundary: one module and period; historical cleaning route and paired route performance unestablished.
MIX production	15–21 February 2023; seven recipe-specific cycles. Evidence: enterprise-derived record.	Role: cycle mass and processing electricity. Combination: MP-06 supplies one same-feed comparison. Boundary: seven recipes are not MP-06 replicates; startup preheating excluded.
Product tests	PE batch properties; three linked MIX specimen groups; independent pallet report. Evidence: enterprise-derived record; reported test methods.	Role: descriptive properties under stated methods. Combination: keep specimen-to-feed links explicit. Boundary: no common numerical PE acceptance specification, MP-06 pallet validation or paired-route functional equivalence.
Factors and economics	Eighteen characterized background factors; cost tariffs and case-study inputs with separate available dates. The 2026 label denotes the economic scenario.	Role: matched climate contributions and named cost scenarios. Combination: common factor snapshot and consistent service/price scope. Boundary: not a common measurement or quotation year, complete environmental assessment or complete business cost.

**Table 2:** Classification criteria and record-based routing interpretations.

Quality State	Joint Composition and Moisture Criteria	Record-Based Routing Interpretation
I	Only one of PE and PP is present in the records; *w* < 1%; impurities < 1%; other polymers = 0.	5 samples; candidates for single-polyolefin reprocessing. The PE equipment requires moisture ≤ 1% and particle sizes of 5–15 mm; PP requires a separate process inventory.
II	Only one of PE and PP is present in the records; *w* < 1%; 1% ≤ impurities ≤ 5%; other polymers = 0.	34 records (PE: 23; PP: 11). II-PE is used in conditional dry/wet scenarios with assumed retained-output shares; product acceptance and route equivalence are not established.
III	PE and PP are both present; *w* ≤ 2.5%; PE + PP ≥ 70%; other polymers < 7%; 10% ≤ paper ≤ 25%.	17 samples. The MIX equipment requires PET ≤ 5%. The same-feedstock comparison uses MIX-06; material specimens were cut from MIX-01, 04, and 07.
IV	PE and PP are both present; *w* > 2.5%; impurities > 5%; PE + PP < 70%; other polymers ≥ 7%.	4 samples; no comparable intensified-treatment route is covered by the available inventories.
U	Unclassified: the rules do not yield a unique assignment, or composition information is insufficient.	All 60 records have a unique class at the 5.0% rule. Table S6 reports classification sensitivity. Technical applicability is recorded separately; unresolved suitability retains all corresponding mass without renormalization.

Counts refer to the 5.0% Class II impurity upper bound. Percentages use wet mass for moisture and dry mass for components. Recorded zero is an operational assignment, not proof of polymer absence. Table S6 summarizes rule scans at 4.5%, 5.0% and 5.5%; sample-level threshold distances are generated in regional_inputs/threshold_distances.csv within the selected output directory. Technical applicability is assessed separately from these record-based classes. PP, polypropylene; PET, polyethylene terephthalate.

**Table 3:** Process quantities and service coverage at the reference coordinates.

Item/Basis (Source and Boundary)	PE Dry	PE Wet	MIX Molding	MIX EB Reference
Functional unit	1 t pellets at *q*_D_ = 1; 1 t assumed retained output after dividing activities by *q*_D_	1 t pellets at *q*_W_ = 1; 1 t assumed retained output after dividing activities by *q*_W_	1 t MIX-06 feed	1 t of the same MIX-06 feed
Observed module	12 post-cleaning pellet batches; enterprise-derived record; pelletizer, cutting, cooling pumps, preheating and standby; cleaning/drying excluded	Same downstream module; transferred cleaning/drying inventory; local meter not available	One MP-06 cycle; enterprise-derived record; processing excludes startup preheating; active processing duration approximately 1–2 h	No MIX-06 extrusion trial; transferred inventory and analyst-defined engineering scenario
Cleaning or reference service	Hypothetical cleaning coordinates; engineering estimate	Literature-transferred services per t final pellets; transferred inventory plus assumed recovery	Startup excluded; additional-service scan is an analyst-defined scenario	rPP extrusion SEC transferred from literature; yield is analyst-defined scenario
Feed coefficient at stated coordinate	1.0810 t dry-film equivalent	1.0755 t dry-film equivalent	1 t incoming feed	1 t incoming feed
Electricity (kWh/FU)	439.8 downstream + 108.1 hypothetical cleaning	439.8 downstream + 481.386 transferred	283.9 recorded processing	311.32 transferred reference
Water (m^3^/FU unless noted)	0.2603 recorded downstream make-up; dry-cleaning water unquantified	2.2603 ordinary water + 0.12663 DI dosing water; 2.159 wastewater sent off site (m^3^/FU)	0.060 assumed	0.080 assumed
Added formulation inputs	No additive/masterbatch reported in the 12 pelletization batches; incoming chemical composition not established	None in pelletization; wet chemicals retained	No separately documented additive input	No matched formulation
Output and residues	1000 kg pellets; 39.2 kg recorded solid residue; 28.0 kg unallocated difference, per t pellets	Same downstream outputs at *q* = 1 and matched quantity; upstream cleaning residues remain unquantified	927.4 kg product; 41.0 kg trim; 12.6 kg rejects; 18.9 kg calculated unallocated difference per t feed	905 kg product assumed; 95 kg remainder fate unknown
Unmatched services	Industrial preparation, sand loss/regeneration, cleaning water and residue climate factor unquantified	Transferred service applicability, upstream residue fate and unmatched disposal billing/climate contributions unresolved	Startup preheating and remaining services outside the recorded meter boundary unquantified	Conditioning, filtration, complete forming/cooling, startup allocation and residue treatment unquantified
Cleaning/sorting and size reduction	Dry-cleaning electricity and recovery are scenarios; preparation scope remains unquantified	Washing/shredding: 240.0 kWh/t pellets, transferred and included	Feed preparation before the measured cycle unquantified	Conditioning and filtration unquantified
Drying	Excluded from local meter; industrial dry-route requirement unresolved	182.4 kWh/t pellets, transferred and included in principal boundary	No separate production-drying quantity established	No matched drying service
Pelletization/compounding	439.8 kWh/t pellets, recorded downstream module	Same downstream module at equal *q* and quantity	Included in 283.9 kWh/t feed processing record	311.32 kWh/t feed, transferred rPP extrusion × assumed yield
Forming/cooling and startup	Cutting/cooling, preheating and standby included in downstream electricity	Same downstream boundary; no double counting of drying	Pressing/cooling within the recorded cycle included; startup preheating excluded; additional services outside the cycle unquantified	Complete board forming/cooling and startup unquantified
Dosing water and chemicals	No recorded cleaning chemicals; industrial services unresolved	126.63 kg DI water; 8.1 kg PAC, 24 kg 50% NaOH solution, 0.27 kg PAM. Climate matched; DI purchase unpriced	No separately documented formulation input	No matched formulation input
Solid residues and non-retained product	Recorded residue: 400 CNY/t bundled charge included; climate factor unavailable. *q*-related product treatment unquantified	Shared residue cancels only at equal activity/*q*; upstream residues unquantified	Trim/rejects transferred free; no displacement credit; downstream burden unknown	95 kg/t feed non-product at reference yield; fate and burden unknown
Wastewater	Industrial dry-cleaning wastewater unresolved	58.986 kWh/t onsite treatment and 2.159 m^3^/t offsite service included sequentially; unmatched settlement/sludge treatment unresolved	No measured whole-line balance	No measured whole-line balance
Sand loss and regeneration	Industrial quantities unquantified; laboratory reuse does not define industrial intensity	No matched sand service	No matched sand service	No matched sand service
Transport	Separate dry-film-equivalent coefficient × distance; not wet mass without moisture	Separate dry-film-equivalent coefficient × distance	1 t common feed; relative distance varied	1 t same feed; common segments cancel
Capital and procurement	L2 annualized by pellet output; procurement scenario uses *μ*_D_	L2 annualized by pellet output; procurement scenario uses *μ*_W_	L2 annualized by feed throughput; common feed price cancels only in difference	L2 annualized by feed throughput; different purchase price retained in difference

FU denotes functional unit; SEC, specific electricity consumption; DI, deionized water; PAC, polyaluminum chloride; PAM, polyacrylamide. PE uses *e*_D_ = 100 kWh/t dry cleaning feed, *r*_D_ = 0.98, *r*_W_ = 0.985 and *q*_D_ = *q*_W_ = 1; route quantities are divided by the affected *q* in retained-output scenarios. MIX uses one tonne of incoming MIX-06 feed. “Recorded” identifies an enterprise-derived value, “transferred” a literature inventory, “scenario” an assumed input and “unquantified” an unavailable contribution. Shared PE services cancel only at the same activity quantity and *q*. Ordinary water, deionized dosing water and wastewater are distinct physical inventory flows. Chemical purchasing uses 24 kg of 50% NaOH solution, whereas its matched NaOH background activity is 12 kg active substance. L2 includes priced direct inputs, labor, maintenance and annualized equipment costs.

**Table 4:** Conditional route differences and transport-crossing states.

Comparison	Settings	Difference or Crossing; Sign, Root Status and Condition
PE cleaning-electricity equality	*r*_D_ = 0.98; *r*_W_ = 0.985; *q*_D_ = *q*_W_ = 1; full wet drying	485.2 kWh/t dry feed for covered climate; 468.8 for covered L2 cost. Positive equality coordinates; wet−dry is positive below each root and negative above it under the stated settings.
PE low electricity coordinate	*e*_D_ = 100 kWh/t dry feed; same recovery and *q*	Wet minus dry: 356.8 kg CO_2_-eq/t pellets; CNY 326.9/t. Positive starting margin at *e*_D_ = 100; direction is toward zero as *e*_D_ increases.
PE high electricity coordinate	*e*_D_ = 500 kWh/t; other settings unchanged	Negative starting margins: −13.7 kg CO_2_-eq/t and −27.7 CNY/t; no positive loss-of-advantage crossing in the tested domain. Dry is not advantaged at this coordinate.
MIX extra transport	*e*_EB_ = 0.344 kWh/kg product; y_EB_ = 0.905; Δ*d* = *d*_MP_ − *d*_EB_; same feed and freight service	Climate root 150.5 km; L2 root 34.0 km. Both start positive at Δ*d* = 0 and decline with increasing relative molding distance. Shared baseline transport cancels.
MIX extra electricity	Other reference settings fixed; *h* = MP_extra_ − EB_extra_ in kWh/t feed	Climate root 27.4 kWh/t feed; L2 root 24.9. Both start positive at *h* = 0 and decline with added net molding electricity. These are margins, not measured omitted demand.
Regional deployment	Seven states; ten annual proxies; fixed feed and equal output	Prescribed adoption may be infeasible under capacity or zero-extra-feed constraints; no single root is implied. Endpoint-specific results and feasibility statuses are retained in Section S8.
MIX net extra electricity *h* = 20	Same reference coordinate; Δ*d* varied	Climate distance root 40.8 km; L2 root 6.7 km. At *h* = 40 or 80, both indicators are already unfavorable at Δ*d* = 0, so no positive loss-of-advantage crossing exists.

Differences are reference minus first route. MIX distance means *d*_MP_ − *d*_EB_ at equal one-tonne feed and matched baseline transport, rather than either route’s total length. PE coefficients use the stated dry-film-equivalent basis. Positive roots are reported with their direction; nonpositive roots are retained as algebraic results without converting them to positive distances. All equalities concern covered services and the stated engineering and price assumptions.

## Data Availability

Anonymized enterprise, flow and composition records, production data, foreground activities, the characterized-factor snapshot, scenario inputs, calculation code and figure-source data are provided in the Supplementary Files. Table S1 documents source coverage; Tables S3, S7–S9 and S11 and the associated registers identify additional evidence, checks and unavailable fields. reproduce_all.py is the calculation entry point, with README.md and output_mapping.csv specifying commands and result locations. The proprietary ecoinvent database is not redistributed. Confidential original enterprise ledgers are not included in the public archive. Original equipment quotations, their dates and itemized scope are unavailable. Original PE electricity start/end readings and feed-moisture weighing pairs are not available in the supplied archive. Computational checks reproduce the disclosed foreground arithmetic and conditional analyses without independently validating unavailable source records.

## Supplementary Materials

The following supporting information can be downloaded at: https://www.mdpi.com/article/10.3390/polym18212646/s1, Supplementary_Materials_Revised PDF (Sections S1–S10, Tables S1–S12 and Figures S1 and S2); Supporting_Data_S1–S8.xlsx; Economic_Parameter_Sources.xlsx; Supplementary_Code.zip (disclosed inputs, executable calculations, figure source data and validation outputs). Figures are embedded in the manuscript; editable vector and high-resolution figure files are supplied separately.
