# Supplementary material for: Conditional Climate–Cost Trade-Offs and Transport Break-Even Conditions for Waste Flexible-Film Recycling Under Feedstock Quality Constraints
Source: Polymers (Basel). 2026 Oct 25;18(21):2646. doi: 10.3390/polym18212646 (PMC13766152; doi:10.3390/polym18212646)
Supplement: Supplementary file 1 [file polymers-18-02646-s001.zip › Supplementary_Materials_Revised.pdf]

Supplementary Materials
Conditional Climate–Cost Trade-offs and Transport Break-even Conditions for Waste Flexible-Film Recycling under Feedstock Quality Constraints

These materials document regional handling records, operational composition classes, route evidence, process inventories, background factors and conditional calculations. Tables S1, S3, S7–S9 and S11 document provenance and evidence availability, batch statistics, reporting-resolution scenarios, procurement costs and characterized-factor details. Supporting_Data_S1-S8.xlsx contains the corresponding data blocks; Economic_Parameter_Sources.xlsx records economic source status. Supplementary_Code.zip contains the executable inputs, code, figure source data and validation outputs. Citation numbers and the functional units for polyethylene (PE) and mixed feed (MIX) follow the main text.

Supplementary data index

Dataset	Main contents	Records or analytical scope
S1	Enterprise reporting-period totals, flows, and regional fields	52 enterprises; 166 flows; 624 underlying monthly records
S2	Sample masses, composition, and joint-rule assignments	60 samples; 15 enterprises × 4 periods
S3	Route applicability and supporting evidence	Equipment inlet requirements, engineering records, and literature
S4	PE batches, material flows, and product tests	12 batches; retained PE product tests
S5	MIX composition, molding, material tests, and load-bearing report	30 material specimens (15 linked; 15 unlinked); 30 linked tensile/flexural measurements; independent load-bearing report
S6	Foreground inventories, background factors, mapping, and engineering costs	Eighteen exported factors; mapped activities, unknown-burden register and tiered costs
S7	Process scenarios, transport thresholds, and unknown net-burden thresholds	5566 route scenarios; equality roots, distance statuses and unknown-burden allowances
S8	Regional matching endpoints and robustness	Seven states; ten annual-quality proxies; adoption, capacity and supply constraints

PE, polyethylene; MIX, mixed feed.

S1 Regional records and field definitions

Monthly and material-flow records are linked through anonymized enterprise identifiers for September 2020 to August 2021. The 624 enterprise–month entries and 166 flow records reconcile to 68,096 t. Transport activity is 16,826,473.2567 t·km; external transfers span 9.4–761.1 km at recorded 0.1 km resolution. Checks found no duplicate enterprise–month keys, flow IDs, complete flow records after excluding flow ID, or enterprise–campaign keys (Regional_Record_Checks.csv). These checks do not identify the same physical material after resale. Without transaction and batch links, neither a non-overlapping intervention subset nor its coverage can be verified. The quantities and regional allocations describe recorded handling, not unique regional waste generation. Table S1 documents coverage and temporal transfer; no composition is imputed to the 37 enterprises without composition observations.

S2 Composition calculations and operational quality classes

S2_Composition_Classes contains compiled wet, dry and component-mass records for 60 samples from 15 enterprises across four campaigns. Moisture uses wet mass and components use dry mass. At the 5.0% Class II impurity bound, Classes I, II, III and IV contain 5, 34, 17 and 4 records; Class II includes 23 II-PE and 11 II-PP records; PP denotes polypropylene. The reported identification procedure used incoming-material information and portable infrared (IR) spectroscopy (IR2200; Optosky Photonics Inc., Xiamen, China). Paper, mineral matter and oil-associated material were operationally separated categories; other-impurity mass was calculated as dry mass minus the seven listed component masses. Sample-specific weighing sheets, spectra and regional drying-condition records are unavailable in the retained dataset (Table S3). The PE drying procedure in S4 is not assigned to these regional samples. A recorded zero does not establish analytical absence or a detection limit, and closure using a residual category does not validate recovery. The seven-state model retains U for unresolved classification; technical applicability is a separate evidence layer. These are study decision rules, not verified industrial quality grades.

S3 Route applicability and supporting evidence

Route screening combines record-based quality, equipment inlet requirements and the required processing service. Table S5 distinguishes documented equipment conditions, observed processing and literature-transferred or assumed services. Complete source details are provided in S3_Route_Evidence. Classification alone does not establish removable surface contamination, numerical product equivalence or the availability of a complete treatment line.

S4 PE batch inventories and product tests

S4_PE_Processes_Products reports twelve production batches processed on 2–13 November 2024. The retained batch records, as confirmed by the authors, indicate that no external additive or color masterbatch was introduced; the predominantly white waste film was weighed after cleaning and necessary drying, immediately before pelletization. Source IDs PE-ADD-01–PE-ADD-12 are formulation-source identifiers for the no-additive batch statements, not records of added materials. The retained records do not identify the historical cleaning route. Using the specified integer-kilogram masses and integer-kWh inputs, totals are 40,796 kg feed, 38,226 kg pellets, 16,811 kWh and 9.95 m^3 make-up water. The pooled apparent output ratio is 93.7%; electricity is 439.779 kWh/t pellets or 412.075 kWh/t recorded feed. Electricity covers pelletizing, cutting and cooling auxiliaries, including batch preheating and standby, but excludes cleaning and drying. Source IDs PE-ELEC-01–PE-ELEC-12 identify the retained batch-ledger entries at 1 kWh resolution; starting and ending readings are unavailable in the retained study dataset. Batch masses and electricity values are enterprise-derived records.

The batch mass account comprises 38,226 kg pellets, 1497.8 kg recorded combined solid residue and a 1072.2 kg unallocated difference, representing 93.700%, 3.671% and 2.628% of feed (Table S7). The residue excludes discarded metal screens and is reported as externally incinerated general solid waste; its composition is unknown. The 400 CNY/t bundled charge covers collection, loading and incineration once. Recorded inlet gravimetric fractions correspond to 299.524 kg; assigning all of this to the difference would still leave 772.676 kg unresolved. This diagnostic does not identify actual water, volatile, stock or other losses. The reported PE moisture procedure was 60 °C for 4 h, 30 min desiccator cooling, then successive 1 h drying cycles until mass change was below 0.1% of sample mass; the original paired masses are unavailable. Ash follows GB/T 9345.1-2008 method A [45] with pre-charring and 600 ± 25 °C ignition; melt mass-flow rate (MFR) uses 190 °C/2.16 kg and GB/T 3682.1-2018 [44]; tensile testing follows the reported GB/T 1040.2-2022 conditions [46]. No specimen count is inferred. Density and specimen-geometry records are unavailable. Customer use concerned a 30 wt% recyclate blend for heavy-duty packaging film; numerical common acceptance limits are unavailable, and injection-molded test specimens do not establish final-film equivalence.

The case pelletizer specifies particles of 5–15 mm and inlet moisture ≤1%. Industrial cleaning electricity, dry-mass recovery and qualification shares are explicit analytical coordinates. The laboratory records do not provide an industrial cleaning electricity inventory, complete metering boundary or validated continuous laboratory-to-production linkage. The laboratory records include ten entries labelled as reuse tests and three labelled as post-heating tests; the reported heating conditions were 600 °C for 5 h. Industrial media loss and regeneration requirements remain unquantified. The pulsed gas–solid cleaning platform was built by the authors in Shanghai, China. Sand heating used an STR SBF200 fluidized sand-bath furnace (Luoyang Saiterui Intelligent Equipment Co., Ltd., Luoyang, China).

S5 Mixed-feed inputs molding cycles and product evidence

S5_MIX_Cycles_Products contains seven distinct formulation/cycle records dated 15–21 February 2023, not seven MIX-06 replicates. Incoming stocks total 3743.4 kg; the seven tested cycles processed 213.7 kg, produced 197.2 kg and consumed 54 kWh. Masses are retained at 0.1 kg resolution and electricity at integer-kWh resolution; neither is a verified instrument-accuracy specification. MIX-06 stock was 626.2 kg, whereas MP-06 processed 31.7 kg, produced 29.4 kg and used 9 kWh: 283.9 kWh/t feed or 306.1 kWh/t product. EX-M1, EX-M2 and EX-M3 each contain five technical specimens linked to MIX-01, MIX-04 and MIX-07. The sheet also retains three unlinked five-specimen groups (15 records) without feed or cycle assignments; these records are not used in Table S12 or for route-equivalence inference. The independent pallet report dated 28 February 2023 records terminal fork-lifting and stacking loads of 1012 and 4998 kgf, with one item per test. It has no traceable MP-06 batch link; terminal loads are not rated capacity, lifetime or evidence of route-level product equivalence. The cycle and specimen records are enterprise-derived

records; the transferred extrusion-based engineering reference (EB) and wet-route inputs are identified separately in S6.

The recorded MP-06 cycle electricity includes twin-screw compounding and heating, melt pumping or metering, pressing, cooling and associated auxiliaries within that cycle. Additional forming or cooling services outside the recorded cycle are unquantified. It excludes startup preheating; active processing duration was approximately 1–2 h. Additional electricity of 0, 20, 40 and 80 kWh/t feed is tested independently for molding and EB as a service-boundary stress test, not measured preheating demand. The equipment requirement of polyethylene terephthalate (PET) ≤5% remains separate from the regional Class III rule for other polymers. Sample preparation for composition analysis is not production pretreatment.

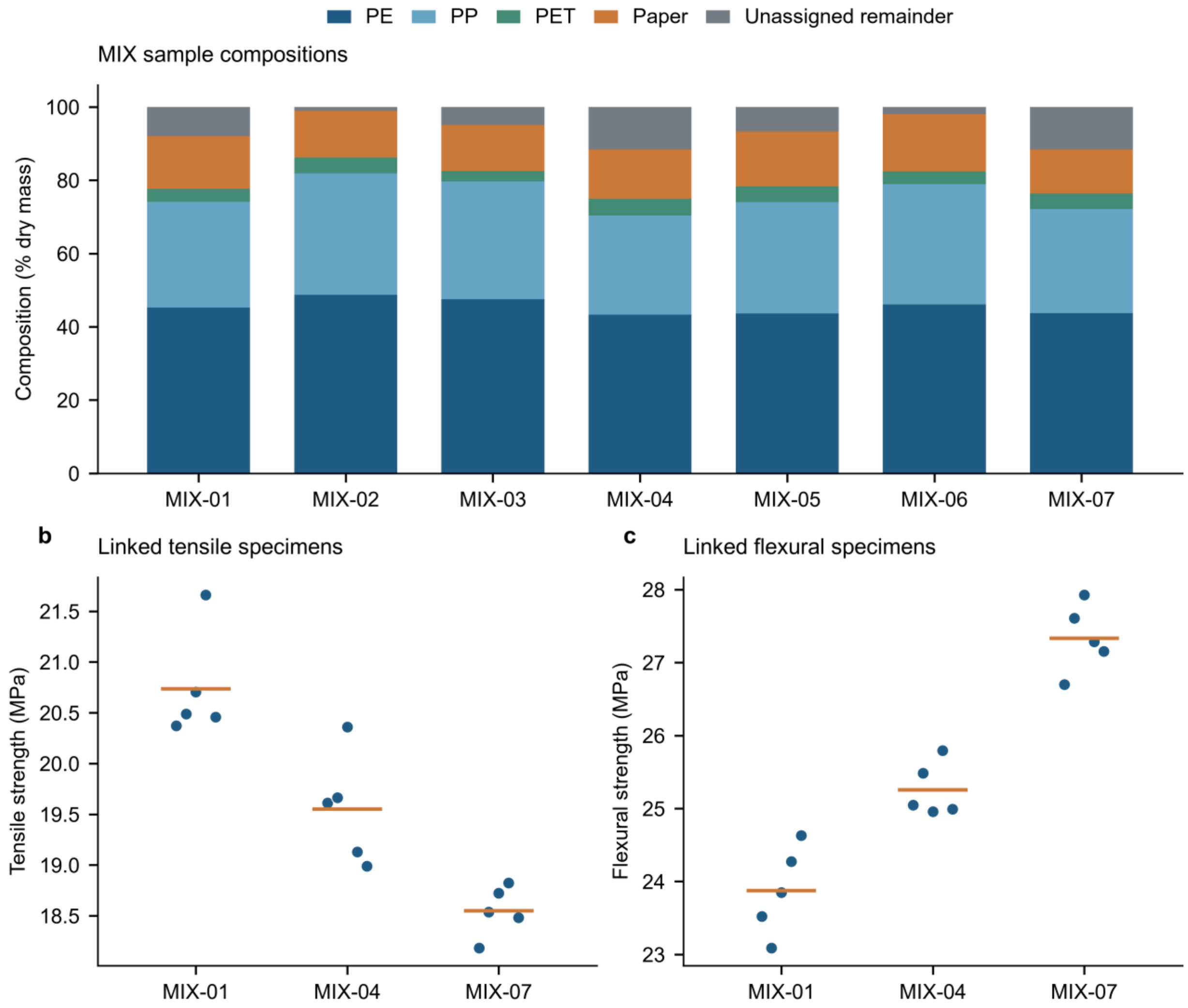

Figure S1. MIX composition and linked material-specimen records. (a) Dry-mass composition; the unassigned remainder is calculated by difference and is not identified as paper or a known impurity. (b,c) All five technical specimens in each of EX-M1, EX-M2 and EX-M3, linked to MIX-01, MIX-04 and MIX-07. Orange lines denote within-group means; no inferential comparison is made. These groups do not validate MP-06 or establish equivalent final-product service. PE, polyethylene; PP, polypropylene; PET, polyethylene terephthalate; MIX, mixed feed.

MIX dry-mass composition lists PE, PP, PET and paper, identified using incoming-material records, portable infrared spectroscopy (IR2200-RT; Optosky Photonics Inc., Xiamen, China) and separation/weighing. The unassigned remainder is 100% minus these four components, with unknown composition. Moisture and ash are separate measurements and are not added to the dry-component sum. MIX-06 contains 46.1% PE, 32.8% PP, 3.5% PET, 15.6% paper and 2.0% unassigned remainder; moisture is 2.02% and ash 5.96%. Moisture and ash use the PE procedures above. Composition specimens and molding feed came from the same homogenized batch. Paper fractions for EX-M1–EX-M3 are 14.4%, 13.5% and 12.0%, so group order is not an increasing-paper series. Trim and rejects were weighed separately and transferred free of charge to an external recycler, without on-site return or a displacement credit. Their zero transaction price does not establish zero downstream burden.

S6 Foreground inventories background processes and economic parameters

S6_Inventory_Factors_Costs identifies foreground activities, matched background processes and eighteen characterized factors. The factors were exported from SimaPro 10.1.0.4 using ecoinvent 3.10 [48], allocation, cut-off by classification, system-process datasets, and the Intergovernmental Panel on Climate Change (IPCC) 2021 100-year global warming potential (GWP100) method [49], V1.03 [50]. Fossil, biogenic and land-transformation components are summed without normalization or weighting [51]. Process names, universally unique identifiers (UUIDs), geographic scopes, reference units and conversions remain documented. East China medium- and low-voltage electricity factors are 0.85680724 and 0.88383483 kg CO_2-eq/kWh; the reference freight proxy is 0.15620259 kg CO_2-eq/(t·km). The public calculation recombines exported factors with foreground quantities. It does not rebuild the proprietary background

network or constitute a new SimaPro run. No applicable PE solid-residue incineration factor occurs in the eighteen-factor export; unknown mixed-residue composition precludes substituting a pure-PE factor. Covered climate subtotals exclude unquantified services and virgin-production credits. Water is reported as a physical input, not a scarcity-weighted water footprint. Exported characterization factors are transferred inventories; price coordinates and auxiliary sensitivities are analyst-defined scenarios.

Capital uses a 10-year life, 6% discount rate and 5% residual value. The PE dry- and wet-route investment inputs are CNY 2.61 million and CNY 2.94 million, respectively, as reported in the compiled enterprise case data.

MIX capital inputs of CNY 8.50 million for molding and CNY 7.20 million for EB are enterprise-reported case-study inputs. Original quotations, their dates and itemized scope are unavailable for independent verification, so equipment-scope equivalence is not established for either comparison. The year 2026 denotes the comparison scenario, not a recovered quotation date. These inputs and the other economic entries are case-study cost assumptions, not independently verified transaction prices.

The chemical-purchasing scenario prices 32.37 kg of supplied products at CNY 2.60/kg, including 24 kg of 50% NaOH solution, 8.1 kg polyaluminum chloride (PAC) and 0.27 kg polyacrylamide (PAM). The NaOH background quantity is 12 kg active substance, with a separate solvent-water test for the other 12 kg. Wet-route source inventories [26] supply 240 kWh washing/crushing, 182.4 kWh drying and 58.986 kWh on-site water treatment per tonne of pellets, plus 2 m^3 ordinary water, 126.63 kg deionized dosing water and 2.159 m^3 effluent. On-site treatment precedes off-site wastewater treatment; these are not duplicate services. Source extrusion and pelletization electricity is replaced by the local module. Unpriced deionized-water and unmatched effluent charges remain unknown. The illustrative molded-product and board prices are CNY 2350 and 3000/t product, not observed transactions or 2026 market averages.

Equipment-price source status and unmatched scope are recorded in Economic_Parameter_Sources.xlsx and Table S11.

Capital sensitivity changes the explicit throughput and utilization denominator without combining the single-line case-study inputs with a 10,000 t/year capacity. A larger deployment requires its own investment, service scope and capacity assumptions; it is not an observed scale-up of this line. Fixed-feed and equal-output regional calculations retain the applicable single-line capacity and additional-supply constraints.

S7 Engineering scenarios and thresholds

The 5566 route scenarios include 252 PE engineering/retained-output combinations, 32 PE background combinations, 64 MIX background combinations and 5184 MIX engineering combinations, with separate utilization, price, shipping and omitted-service families. They are finite parameter combinations, not a probability distribution. MP denotes the molding route; EB-minus-MP climate differences span −58.197 to 129.670 kg CO_2-eq/t feed across MIX engineering scenarios; the corresponding L2 range is −51.873 to 108.641 CNY/t feed. Background/auxiliary scenarios span 23.461–26.438 kg CO_2-eq/t, and the 16 two-sided additional-electricity scenarios span −45.032 to 92.057 kg CO_2-eq/t. The EB reference of 0.344 kWh/kg product is the reported average for recycled-PP extrusion [32], including motor and barrel heating. It is not a MIX-06 board-line measurement. Yield 0.905 and ranges 0.254–0.450 kWh/kg and 0.88–0.93 are engineering assumptions; conversion gives 311.32 kWh/t feed at the reference coordinate.

At the MIX reference coordinate, accounted climate impacts are 243.340 kg CO_2-eq/t feed for MP and 266.852 for EB; L2 costs are 972.765 and 993.187 CNY/t feed. Their differences, 23.512140 kg CO_2-eq/t and 20.421804 CNY/t, are net omitted-burden allowances with other terms fixed, not estimates of omitted services. Using the same feed and freight service, relative MP distance removes these margins at 150.523369 and 34.036340 km. Net additional MP electricity removes them at 27.441575 and 24.904639 kWh/t feed. Roots retain their signs and crossing status. For PE at $r_D = 0.98$, $r_W = 0.985$ and $q_D = q_W = 1$, dry-cleaning equality coordinates are 485.208 and 468.805 kWh/t dry-film equivalent for climate and L2; these do not establish achievable industrial demand. The corresponding EB electricity equality coordinates are 0.313677817 kWh/kg product for climate and 0.316481062 for L2 cost.

At assumed product prices of 2350 CNY/t MP product and 3000 CNY/t EB product, processing cost less revenue is −1206.730 and −1721.813 CNY/t feed. These figures omit feed purchases and unpriced services and are not profit estimates. Product-price equality holds other covered terms and yields fixed and gives no value or displacement credit to freely transferred residue. Procurement terms and their non-cancellation for unequal PE feed demand are specified in Table S11 and Equations

(S12a)–(S12c). EB is lower by 515.082928 CNY/t feed on this processing-cost-less-revenue basis; the corresponding board-price equality is 2430.847593 CNY/t product.

S8 Joint quality and flow matching and robustness

The regional model allocates seven quality states across all 53 flows from the fifteen sampled enterprises: 371 nonnegative variables and 158 equality rows, including redundant conservation equations. Each climate or cost endpoint is optimized independently, with its mass, transport work, output and additional feed taken from that same solution. Ten annual-quality proxies comprise equal-frequency weights, monthly-throughput weights, four single-campaign proxies and four leave-one-out proxies. The monthly proxy assigns each month to the nearest sampled month, splitting November equally between October and December; this extrapolates observed composition, not monthly composition measurement. Only recorded washing-to-pellet flows assigned to II-PE are candidate interventions. Transaction-level traceability is insufficient to verify a deduplicated intervention subset, whose coverage is unavailable rather than zero. Results therefore describe conditional record-based allocations.

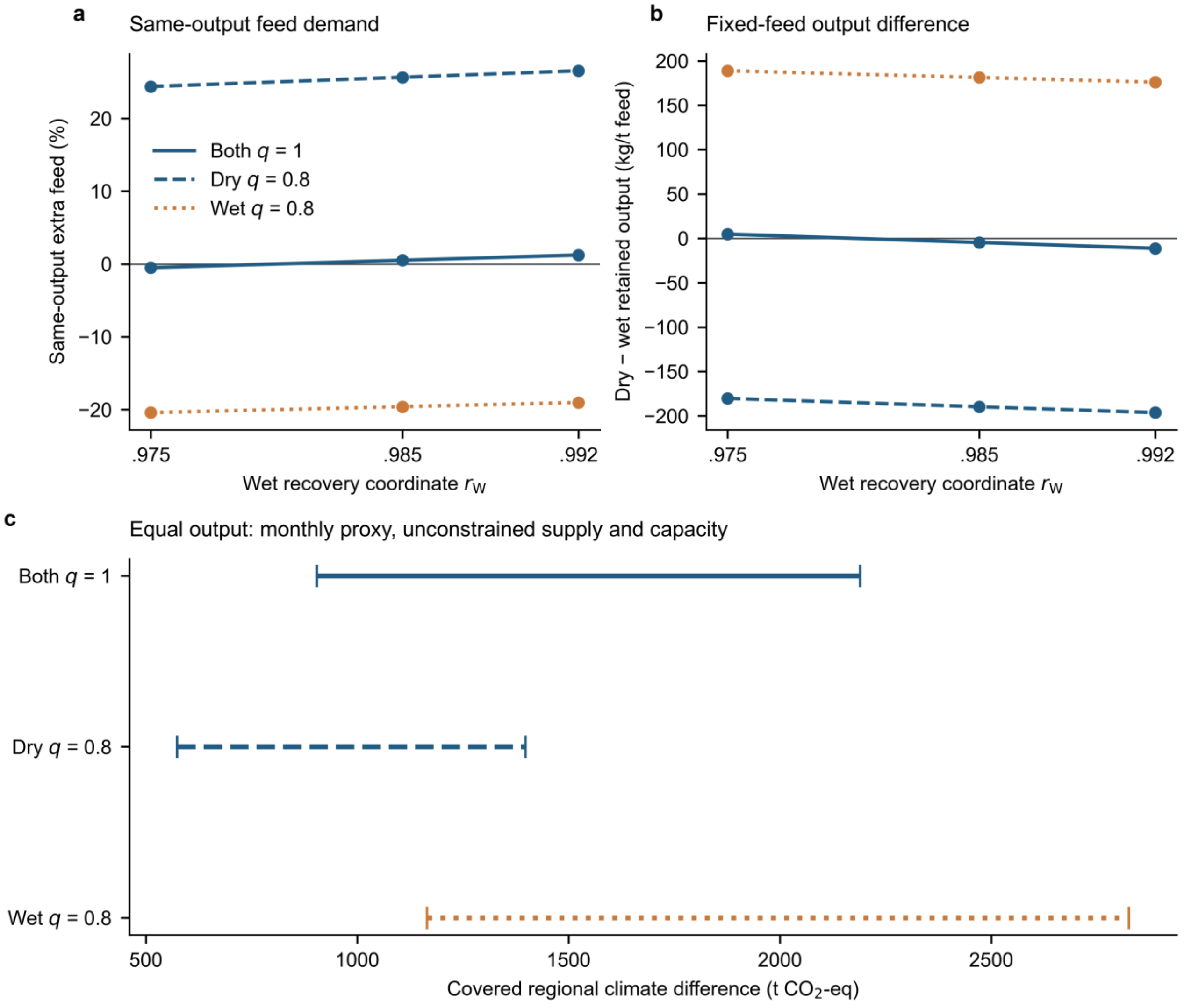

Figure S2. Mass-demand consequences of recovery and q assumptions. (a) Additional dry-route feed for equal retained pellet output and (b) retained-output differences at fixed feed, using the reported mass coefficient, r_D = 0.98 and the indicated wet recoveries. (c) Independently solved equal-output regional climate endpoints for the monthly proxy at e_D = 100 kWh/t dry feed, r_W = 0.985, full adoption and no capacity or additional-feed ceiling. The q settings are analytical stress tests, not measured grade acceptance. Negative additional feed in (a) denotes lower feed demand under that setting; no substitution credit is assigned to output differences.

S8.1 Additional sensitivity to wet-route recovery

Wet cleaning dry-mass recovery is not directly transferable from the product-normalized source inventory. Values of 0.975, 0.985 and 0.992 are analytical linkage assumptions. The dry pelletization-feed coefficient is b = 1.059396 t dry film/t pellets, from the retained feed-moisture percentages and batch totals. For route R, the conditional incoming requirement is $\mu_R = b/(r_R\, q_R)$ tonnes of dry-film equivalent per tonne of qualifying pellets. Incoming wet tonnes additionally require route-specific moisture; regional recorded tonnes are used as dry-film-equivalent tonnes only under a declared scenario conversion.

Changing wet recovery changes feed demand, transport and regional conversion; it does not rescale source services already expressed per tonne of final pellets [26]. PE capital retains its output denominator. The principal boundary keeps all 182.4 kWh/t of wet drying because the local pelletization meter excludes drying. Deductions of 91.2 and 182.4 kWh/t are counterfactual service-overlap tests, not demonstrated corrections. Qualification shares of 1.00, 0.95, 0.90 and 0.80 scale route activities and feed requirements per qualifying output; either route is stressed separately while the comparator remains at 1.00.

At dry recovery 0.98 and equal qualification shares, dry-minus-wet incoming feed per tonne of baseline wet feed is −0.005102, 0.005102 or 0.012245 for wet recoveries 0.975, 0.985 or 0.992. These coefficients follow $r_W/r_D - 1$ and are not invariant route properties. Equal-output scenarios constrain positive extra feed separately from output capacity; zero extra supply can exclude an otherwise beneficial intervention. Fixed-feed scenarios instead permit different outputs, without a credit for their difference.

For common-distance transport, the sign of $\mu_D - \mu_W$ determines whether increasing distance erodes or increases the covered dry-route advantage. With the preceding equal-qualification coordinates, that sign changes between wet recoveries 0.975 and 0.985. An extra-distance test is different: it increases only dry-route distance while holding wet-route distance fixed. A positive intersection exists only when the intercept and slope permit it. Crossings beyond the recorded external-distance range are extrapolations, not validated diversion radii.

S8.2 Data sharing with prior work and the published companion paper

This study reuses the gas–solid cleaning apparatus formally published by Jin et al. in 2025 [42], DOI: 10.1007/s10163-024-02111-5. One of the authors led the design and construction of the platform. Mechanism diagrams, parameter-response curves, and model-film cleaning results from that prior work are not presented as new observations here. Reuse of the apparatus and reuse of experimental records are disclosed separately.

The companion paper, "Film Recovery Constraints on Optical and Gravimetric Assessment of Pulsed Gas–Solid Cleaning of Model Plastic Films" (Polymers 2026, 18, 2593; https://doi.org/10.3390/polym18202593) [43], reports terminal-wash mass-change ratios (R_d) for three laboratory subsamples from each of the twelve waste-PE source batches. Its optical, gravimetric and film-count analyses concern a separate set of 84 model-film runs. It contains no pelletization input/output, electricity or mechanical-property series shared with this manuscript, which uses separately documented production and inventory records. Its R_d values are terminal-wash mass-change endpoints. Table S2 records the distinct laboratory and production uses of the common material sources; these do not establish a continuous cleaning–pelletization experiment.

S8.3 Computational implementation and independent verification

The public process calculation multiplies activities by their compatible exported factors, including the documented 3.6 MJ/kWh conversion where required. Historical SimaPro foreground comparisons support the original export provenance, not an independent rerun of each current route. Component-ledger sums, mass and electricity totals, representative unit conversions, costs and root logic are checked independently from the calculation outputs. Unmapped burdens are retained in a separate register rather than assigned zero. Regional checks test conservation, global constraints and objective values; an enterprise-wise greedy method cannot verify a model with shared capacity or supply limits.

The numerical results were reproduced using CPython 3.12.10, NumPy 2.4.6 and SciPy 1.18.0 with scipy.optimize.linprog (HiGHS); the runtime record identifies the solver build. The entry point writes runtime versions, hashes, arithmetic and feasibility checks, and output mappings. The archive includes selected run summaries and validation outputs, while the full endpoint grid and allocation witnesses can be generated with reproduce_all.py. The proprietary ecoinvent database is not redistributed. The checks establish computational consistency of the disclosed inputs, not independent measurement validation or a new database run.

Run reproduce_all.py from the extracted code directory with a new output directory. It generates route_scenarios.csv, component_ledger.csv, electricity and distance roots, unknown-burden registers, sample classifications, annual proxies, full regional endpoints and allocation witnesses. revision_checks.py and revision_extensions.py add batch statistics, reporting-resolution bounds, procurement scans, signed crossings and service registers. revision_evidence contains selected public tables;

output_mapping.csv and workbook_layout.json identify their table, figure and workbook destinations. Input hashes and numerical workbook/output comparisons are recorded separately. Numerical comparisons use max(10^{-8}, 10^{-8} × absolute reference value).

S8.4 Local sensitivity to the study-defined Class II impurity boundary

The Class II non-plastic-impurity upper bound is scanned at 4.5%, 5.0% and 5.5%, keeping its other criteria unchanged: one of PE or PP, moisture below 1%, impurities at least 1%, and no assigned other polymers. The upper bound is inclusive. Class I, III and IV rules remain unchanged. All seven states, including U, remain in every allocation. Distances from each sample to the relevant thresholds are retained; without measured uncertainty intervals these are decision-rule scans, not simulations of analytical error. A conservative technical-applicability branch retains unresolved material as U without renormalizing the remaining shares.

For the regional grid, each PE engineering/quality case and utilization case regenerates the objective before optimization. Background-factor and service-boundary tests are evaluated at the process level and are not fully crossed with the regional allocation grid. The principal regional comparison fixes feed; the equal-output branch additionally tests extra-supply availability. Adoption is 0, 0.25, 0.50 or 1.00. The nominal single-line output cap is 3072 t/year, with utilization and multi-line assumptions identified separately. Climate and cost endpoints can select different allocations. The 2026 cost scenario is applied to physical records from their stated dates; it is not a reconstruction of 2020–2021 business performance.

The seven-state formulation enforces flow and enterprise/state equalities directly, including when U has positive mass. Neither unassigned flow mass nor technically unresolved material is forced into a recycling class. Added global capacity and extra-supply constraints are tested with the endpoint solution. Infeasible cases retain their status, and adoption zero is included as a no-intervention scenario. A verified intervention-subset result cannot be inferred from record-based feasibility.

At the 4.5% bound, seven II-PE and two II-PP observations move to U; the equal-frequency U proxy mass is 2945.697 t. Raising the bound from 5.0% to 5.5% changes no record-based classifications. In the conservative technical-applicability layer, all 21,617.997 t remain U because the required route suitability is unconfirmed; this does not establish that the material is unsuitable. Table S6 separates rule sensitivity from annual-proxy and technical-applicability analyses. A positive covered record-based endpoint is not observed industrial adoption.

S9 Equations and symbol definitions

Equations (S1)–(S20) define composition, normalization, costs, logistics and regional allocation. Principal symbols and units are listed in Table S4. PE is expressed per tonne of retained pellet output under an assumed q; numerical common acceptance limits are unavailable, so q is not a measured qualifying rate. MIX treats one tonne of the same feed without assuming equivalent product function. Climate is expressed in kg CO_2-eq per functional unit (FU), and cost in CNY/FU. Covered terms and unknown route contributions remain separate; the supplementary cost expansion is given in Equations (S12a)–(S12c).

$$w_i = \frac{m_{\mathrm{wet},i} - m_{\mathrm{dry},i}}{m_{\mathrm{wet},i}} \times 100\% \tag{S1}$$

Moisture content is the difference between wet mass and constant dry mass divided by wet mass, expressed as a percentage in Equation (S1). Equation (1) in the main text uses a dimensionless fraction, multiplied by 100% only when percentages are reported.

$$c_{\mathrm{imp},i} = \frac{m_{\mathrm{paper},i} + m_{\mathrm{dust},i} + m_{\mathrm{oil},i} + m_{\mathrm{other\ impurity},i}}{m_{\mathrm{dry},i}} \times 100\% \tag{S2}$$

Non-plastic impurities are the dry-mass sum of paper, mineral matter, oil-associated material and other recorded impurities. The operationally assigned oil category is not an independently validated total-oil assay. Classification and technical route applicability are evaluated separately.

$$\begin{aligned} c_{\mathrm{PO},i} &= \frac{m_{\mathrm{PE},i} + m_{\mathrm{PP},i}}{m_{\mathrm{dry},i}} \times 100\% \\ c_{\mathrm{OP},i} &= \frac{m_{\mathrm{PET},i} + m_{\mathrm{other\ plastic},i}}{m_{\mathrm{dry},i}} \times 100\% \end{aligned} \tag{S3}$$

PO is the sum of PE and PP; OP is the sum of PET and other recorded plastics. Both enter the joint classification rules as dry-mass percentages. Recorded zeros are retained without assigning inferred detection limits.

$$\varepsilon_i = \frac{\sum_k m_{ik} - m_{\mathrm{dry},i}}{m_{\mathrm{dry},i}} \times 100\% \tag{S4}$$

The closure diagnostic compares the sum of component records with dry mass. Because other-impurity mass is calculated by subtraction, closure is partly imposed by construction. It does not establish analytical recovery, instrument precision or independent confirmation of composition.

$$\begin{gathered} M_e = \textstyle\sum_j M_{ej} \\ M_r = \textstyle\sum_f M_f \, 1(\mathrm{route}_f = r) \end{gathered} \tag{S5}$$

Enterprise masses are summed over monthly records, while route masses are summed over retained flow records using route indicators. These totals describe the recorded enterprise network and are not extrapolated to total regional waste generation.

$$\begin{gathered} \pi_{e,s} = \textstyle\sum_t \omega_{e,t} \, 1(c_{e,t} = s), \ \textstyle\sum_t \omega_{e,t} = 1 \\ M_{s,\mathrm{proxy}} = \textstyle\sum_e M_e \, \pi_{e,s} \end{gathered} \tag{S6}$$

For enterprise e, campaign weights ω sum to one. Equal-frequency weights are 1/4; leave-one-out weights are 1/3 for the retained campaigns; a single-campaign proxy gives one campaign weight one. Monthly-throughput weights use September + October + half November; half November + December + January; February + March + April; and May + June + July + August, each divided by annual enterprise mass. The state indicator is determined by the selected classification rule. These ten proxies do not impute quality for the other 37 enterprises.

$$\begin{gathered} Y_{\mathrm{app},j} = \frac{P_j}{F_j} \\ e_{\mathrm{product},j} = 1000 \, \frac{E_j}{P_j} \end{gathered} \tag{S7}$$

For batch j, F_j and P_j are feed and pellets in kg and E_j is kWh. Batch intensities are $e_{P,j} = 1000E_j/P_j$ and $e_{F,j} = 1000E_j/F_j$. The inventory uses $e_{P,\mathrm{pool}} = 1000\sum E_j/\sum P_j$ rather than the arithmetic mean of batch intensities. Apparent yield is a mass ratio, not independently measured polymer recovery. PE meter start/end readings are unavailable. MIX treatment instead uses $1000E/F$; MP-06 gives 1000 × 9/31.7 = 283.911672 kWh/t feed. The factor 1000 converts the kg denominator to tonnes.

$$\begin{gathered} Y_{\mathrm{all}} = \frac{\sum P_j}{\sum F_j}, \ e_{\mathrm{pellets}} = 1000 \, \frac{\sum E_j}{\sum P_j} \\ b = \frac{\sum F_j(1 - w_j)}{\sum P_j}, \ \mu_R = \frac{b}{r_R \, q_R} \end{gathered} \tag{S8}$$

Ratios of PE batch totals give 439.779208 kWh/t pellets and 412.074713 kWh/t recorded feed. The dry-film coefficient b is 1.059396118 t/t pellets. For route R, $\mu_R = b/(r_R \, q_R)$, where r_R is the assumed cleaning recovery and q_R the assumed retained-output share. The affected route activities, impacts, costs and feed demand are divided by q_R; non-retained pellets are $(1-q_R)/q_R$ t per tonne retained, with their unquantified fate explicit. Nominal output capacity is multiplied by q_R once. The original batch masses do not change with these scenarios.

$$\begin{gathered} G_r^{\mathrm{cov}} = \textstyle\sum_{i \in \mathrm{mapped}} A_{ri} \, EF_i \\ G_{\mathrm{route},r} = G_r^{\mathrm{cov}} + G_{\mathrm{T},r}^{\mathrm{cov}} + U_r \end{gathered} \tag{S9}$$

The covered subtotal sums compatible mapped activities and factors. Transport is added separately once. U retains unresolved route-specific services, including PE residue incineration and industrial media regeneration. PE residue destination is known but its applicable climate factor is unavailable. Identical shared services cancel only with the same quantity, condition and functional basis; their burdens need not cancel after unequal qualification scaling.

$$I_{\mathrm{water}} = \frac{V_{\mathrm{makeup}}}{m_{\mathrm{FU}}} \tag{S10}$$

Water intensity is normalized by the declared feed or product mass. m_{FU} is in t, V_{makeup} is in m³, and the resulting I_{water} is in m³/t. Tap-water make-up, deionized water for chemical dosing, and wastewater are reported separately. These are physical inventory quantities, not scarcity-weighted water footprints. Scenario water inputs are distinguished from metered records.

$$\begin{gathered} C_r^{\mathrm{L0,cov}} = \sum_{i\,\in\,\text{priced direct}} A_{ri}\, p_i \\ C_r^{\mathrm{L1,cov}} = C_r^{\mathrm{L0,cov}} + C_{\mathrm{labour},r} + C_{\mathrm{maintenance},r} \\ C_{\mathrm{op},r} = C_r^{\mathrm{L1,cov}} + C_{\mathrm{T},r}^{\mathrm{cov}} + U_r^{\mathrm{op}} \end{gathered} \tag{S11}$$

L0 includes priced direct utilities, materials and treatment services, including the PE residue charge. L1 adds labor and maintenance. Unknown costs remain outside the covered tier. The CNY 400/t residue charge already includes collection, loading and incineration; overlapping services are not added again. Unpriced water-treatment or media services remain unknown, even where other activities in that route are covered.

$$\begin{gathered} C_r^{\mathrm{L2,cov}} = C_r^{\mathrm{L1,cov}} + C_{\mathrm{capital},r} \\ R_{\mathrm{product},r} = \sum_j P_{rj}\, p_{\mathrm{product},j} \\ C_{\mathrm{market},r}^{\mathrm{cov}} = C_r^{\mathrm{L2,cov}} + C_{\mathrm{feed\ purchase},r} - R_{\mathrm{product},r} + C_{\mathrm{T},r}^{\mathrm{cov}} \\ C_{\mathrm{market},r} = C_{\mathrm{market},r}^{\mathrm{cov}} + U_r^{\mathrm{cost}} \end{gathered} \tag{S12}$$

L2 adds annualized equipment capital using annual total pellet output for PE and annual feed throughput for MIX. Utilization is counted once; q is then applied to retained-output normalization. Extended costs additionally require feed purchasing, transport, priced auxiliary services and any separately assumed product revenue. Unknown services remain U_{cost}. Table S11 distinguishes these terms and reports procurement scenarios; no avoided-virgin-production credit is used.

$C_{R,\mathrm{extended}} = C_R^{\mathrm{L2,cov}} + \mu_R\, p_{\mathrm{feed},R} + C_{\mathrm{transport},R} + C_{\mathrm{aux,priced},R} - R_{\mathrm{product},R} + U_{\mathrm{cost},R}$ (S12a)

$\Delta C_{\mathrm{extended}} = C_{\mathrm{B,extended}} - C_{\mathrm{A,extended}}$ (S12b)

$\Delta C_{\mathrm{PE}} = 326.920868 + (1.075529054 - 1.081016447)p_{\mathrm{W}} - 1.081016447\delta p - \Delta U_{\mathrm{cost}}$ (S12c)

$$\begin{gathered} \mathrm{TKM} = \sum_f M_f\, d_f \\ d_{\mathrm{weighted}} = \frac{\sum_f M_f\, d_f}{\sum_f M_f} \end{gathered} \tag{S13}$$

Mass is expressed in t and distance in km. Transport activity is first summed and then divided by the corresponding material mass to obtain a mass-weighted mean distance, with inclusion or exclusion of internal zero-distance records stated. Mean distances for destination groups are not used as the maximum distance of the full sample.

$$\begin{gathered} G_{\mathrm{T},r}^{\mathrm{cov}} = \sum_l \mu_{rl}\, d_{rl}\, EF_{\mathrm{T},rl} \\ C_{\mathrm{T},r}^{\mathrm{cov}} = \sum_l \mu_{rl}\, d_{rl}\, p_{\mathrm{T},rl} \end{gathered} \tag{S14}$$

Each μ denotes transported mass per functional unit for a specified segment. PE uses conditional dry-film-equivalent coefficients unless incoming wet moisture is supplied; MIX uses one tonne of the same feed. The central factor is 0.156202592699214 kg CO_2-eq/(t·km), ID 28198. Vehicle-service descriptions are retained in Table S9; numerical loading and empty-return parameters were not separately extracted, so no extra load multiplier or return ×2 is applied. The central 0.60 CNY/(t·km) tariff and 0.39–0.80 scan are cost scenarios; a return-cost settlement breakdown is unavailable. Only identical segment services cancel.

$$\begin{gathered} \Delta G(D) = A - BD \\ A = H_{\mathrm{B}}^{\mathrm{cov}} - H_{\mathrm{A}}^{\mathrm{cov}} - \Delta U,\ B = (\beta_{\mathrm{A}} - \beta_{\mathrm{B}})f_{\mathrm{T}} \end{gathered} \tag{S15}$$

Route subscripts A and B denote dry/wet for PE and MP/EB for MIX; positive Δ favors A. For MIX, $\Delta G = G_{\mathrm{EB}}^{\mathrm{cov}} - G_{\mathrm{MP}}^{\mathrm{cov}} + \mu_{\mathrm{EB}} f_{\mathrm{EB}} d_{\mathrm{EB}} - \mu_{\mathrm{MP}} f_{\mathrm{MP}} d_{\mathrm{MP}} - \Delta U_{\mathrm{G}}$, and costs use freight prices and ΔU_{C}. Under the same one-tonne feed, factor and baseline transport, $\Delta d = d_{\mathrm{MP}} - d_{\mathrm{EB}}$ gives $\Delta G = 23.512140 - 0.156202592699214\Delta d$ and $\Delta C = 20.421804 - 0.60\Delta d$. Positive Δd is a rerouting scenario relative to a fixed reference, not an observed route disadvantage. Equal-distance transport cancels and negative Δd favors MP. These roots are not validated collection radii.

$$D^* = \frac{A}{B} \quad (B \neq 0) \tag{S16}$$

The distance equality is the signed ratio A/B only if B is nonzero. A positive root and its starting sign determine the crossing direction. $B = 0$ gives no distance dependence; $A = B = 0$ gives equality at all distances. Nonpositive roots are not converted to absolute distances. Distance roots beyond the recorded external range are extrapolations. The same algebra applies to matching covered cost tiers and unknown cost differences.

$$\begin{aligned} e_{\mathrm{D}}^{*} &= \frac{G_{\mathrm{W}}^{\mathrm{cov}} - G_{\mathrm{D,other}} - \Delta U_{\mathrm{PE}}}{\mu_{\mathrm{D}}\, f_{\mathrm{el}}} \\ s_{\mathrm{EB}}^{*} &= \frac{H_{\mathrm{MP}}^{\mathrm{cov}} - H_{\mathrm{EB,nonel}}^{\mathrm{cov}} + \Delta U_{\mathrm{MIX}}}{1000\, \eta_{\mathrm{EB}}\, f_{\mathrm{el}}} \end{aligned} \tag{S17}$$

The PE equality is $e_{\mathrm{D}}^{*} = (G_{\mathrm{W}}^{\mathrm{cov}} - G_{\mathrm{D,other}} - \Delta U)/(\mu_{\mathrm{D}}\, f_{\mathrm{el}})$, with $\mu_{\mathrm{D}} = b/(r_{\mathrm{D}} q_{\mathrm{D}})$; $G_{\mathrm{D,other}}$ excludes only the varied dry-cleaning electricity. $\Delta U = U_{\mathrm{D}} - U_{\mathrm{W}}$ is the net unknown dry-route burden. Cost equality uses matching cost tiers and electricity price, with procurement or priced auxiliary terms included in the other-cost term when present. Laboratory sand reuse and regeneration at 600 °C for 5 h do not locate a full industrial line within this allowance. Preparation, transport within the plant, air service, dust collection, sand losses/regeneration, startup, cooling and residue services are separately registered as unknown where unmetered. In the second equality, s_{EB}^{*} is EB electricity in kWh/kg product, η_{EB} is product mass per feed mass, H denotes covered impact including fixed transport, $H_{\mathrm{EB,nonel}}$ excludes EB electricity, and $\Delta U_{\mathrm{MIX}} = U_{\mathrm{MP}} - U_{\mathrm{EB}}$. The factor 1000 converts tonnes of feed to kilograms. EB electricity equality is 0.313677817 kWh/kg product for climate and 0.316481062 for L2 at the reference setting.

$$\begin{aligned} \textstyle\sum_{s} x_{f,s} = M_{f},\ \sum_{f\in e} x_{f,s} = M_{e}\, \pi_{e,s},\ x_{f,s} \geq 0 \\ \alpha \textstyle\sum_{f} \mathrm{eligible}_{f}\, x_{f,\text{II-PE}}\, o \leq Q_{\mathrm{cap}} \\ \alpha \textstyle\sum_{f} \mathrm{eligible}_{f}\, x_{f,\text{II-PE}}\, v \leq L_{\mathrm{cap}} \end{aligned} \tag{S18}$$

The seven states are I-PE, I-PP, II-PE, II-PP, III, IV and U. The first two equalities conserve every flow and each enterprise/state proxy mass. α is scenario adoption and eligible selects candidate II-PE washing-to-pellet flows. The modeled capacity applies to the dry-route intervention; the wet route is retained as the reference service without a separate additional line-capacity constraint. The output coefficient o is $1/\mu_{\mathrm{D}}$ for fixed feed or $1/\mu_{\mathrm{W}}$ for equal output. Extra-feed coefficient v is zero for fixed feed and $\max(\mu_{\mathrm{D}}/\mu_{\mathrm{W}} - 1, 0)$ for equal output. Q_{cap} constrains scenario quality-adjusted output: nominal total pellet capacity multiplied by q_{D}. Thus the 3072 t/year nominal case has $Q_{\mathrm{cap}} = 3072 q_{\mathrm{D}}$. L_{cap} limits available additional feed. These are deployment assumptions, not observed capacity or measured qualification rates.

$$\begin{aligned} S^{\mathrm{cov}}(x) &= \alpha \textstyle\sum_{f} \mathrm{eligible}_{f}\, x_{f,\text{II-PE}}\, (a + b d_{f}) \\ S(x) &= S^{\mathrm{cov}}(x) - \alpha \textstyle\sum_{f} \mathrm{eligible}_{f}\, x_{f,\text{II-PE}}\, u_{f} \end{aligned} \tag{S19}$$

Positive S favors the dry route within the stated boundary. For fixed feed, $a = G_{\mathrm{W}}^{\mathrm{cov}}/\mu_{\mathrm{W}} - G_{\mathrm{D}}^{\mathrm{cov}}/\mu_{\mathrm{D}}$ and $b = 0$ for identical transport paths; outputs can differ without a displacement credit. For equal output, $a = (G_{\mathrm{W}}^{\mathrm{cov}} - G_{\mathrm{D}}^{\mathrm{cov}})/\mu_{\mathrm{W}}$ and $b = f_{\mathrm{T}}(1 - \mu_{\mathrm{D}}/\mu_{\mathrm{W}})$. Qualified-output scenarios use consistently scaled route totals and μ. Unknown net burdens enter as u per tonne of baseline feed: $U_{\mathrm{D}}/\mu_{\mathrm{D}} - U_{\mathrm{W}}/\mu_{\mathrm{W}}$ for fixed feed, or $(U_{\mathrm{D}} - U_{\mathrm{W}})/\mu_{\mathrm{W}}$ for equal output. Once u is specified, the objective is regenerated and reoptimized. Cost uses the same normalization and sign.

$$S_{\mathrm{low}}^{\mathrm{cov}} = \min_{x\in Q} S^{\mathrm{cov}}(x),\ S_{\mathrm{high}}^{\mathrm{cov}} = \max_{x\in Q} S^{\mathrm{cov}}(x) \tag{S20}$$

Lower and upper endpoints independently optimize the covered objective over the same feasible set, including deployment constraints. Their mass, transport activity, output and additional supply come from their respective solutions, not independently selected extremes. These ranges describe conditional allocations, not confidence intervals or guaranteed savings. Climate and cost extrema need not coincide. Infeasibility and unavailable verified-subset coverage remain explicit, and $\alpha = 0$ represents no intervention.

S10 Additional provenance and supporting evidence

Tables S1, S3, S7–S9 and S11 extend the retained evidence without reconstructing absent original observations. "Unavailable" refers to evidence not provided for the present assessment. Descriptive comparisons and parameter scans retain their stated scope. Full record-level fields are supplied as comma-separated values (CSV) files in revision_evidence and as indexed blocks in the supporting workbook.

Supplementary Tables

Table S1 Data provenance inclusion and coverage

Stage	Retained coverage	Selection evidence and missingness
Registry	64 enterprises identified in the reported registry	Registry construction date, source roster, contact log, district-level coverage and reasons for 12 non-inclusions are unavailable for verification.
Reporting network	52 enterprises; 624 enterprise–month entries; 166 flows	Available-record inclusion; probability-based recruitment cannot be established. The 12 without usable records are not coded as zero.
Composition subset	15 enterprises; 60 records; 53 associated flows	Four observations per enterprise. Selection mechanism is not established. No composition is imputed for the other 37 enterprises.

Observed coverage comparison. Similarity in these observed characteristics does not remove selection bias; no random-sampling confidence intervals are attached.

Group	Firms *n*	Flows *n*	Recorded mass t	Median annual mass t	Weighted distance km
With composition	15	53	21,617.997	1,173.808	245.108
Without composition	37	113	46,478.003	1,094.713	248.025

Dataset and date	Record unit and scope	Role and transfer condition
Network: Sep 2020–Aug 2021	Enterprise month and destination flow; reported Shanghai enterprise network	Defines recorded mass, route and distance. Compiled records survive; physical trade chains are not established.
Composition: Oct/Dec 2020; Mar/Jun 2021	Enterprise–campaign sample from the 15-enterprise subset	Provides annual quality proxies. Sample-level raw weighing/infrared and drying records are unavailable; four observations are not annual composition measurements.
MIX: Feb 2023	Seven formulation/cycle records; one MP-06 cycle	Unit inputs for the same-feed task; not contemporaneous operation of the earlier network.
PE: Nov 2024	Twelve separate pelletization batches at the documented case	Unit pelletization inputs; cleaning-route assignment and meter start/end records are unavailable.
Economics: 2026 reference scenario	Case equipment inputs and parameter prices	Common comparison coordinate; not a recovered common quotation date or historical 2020–2021 cost.

Table S2. Relationship of this study to prior work and the published companion paper.

Object or data	Relationship to prior work or the published companion paper	Use in this manuscript
Apparatus design	Previously published team platform; One of the authors led design and construction.	Documents the method source; the analysis-boundary diagram is redrawn for this study.

Object or data	Relationship to prior work or the published companion paper	Use in this manuscript
Twelve common material-source batches	The published companion paper [43] uses 36 laboratory subsamples; this paper uses separately documented PE production and inventory records. Laboratory subsamples did not enter the production line.	Source correspondence is retained; pelletization mass, electricity and product-test observations are not a shared experimental series in the published companion paper.
Laboratory cleaning endpoints	The published companion paper [43] analyzes optical, gravimetric and film-count endpoints. Published apparatus research [42] is a separate source.	Neither source establishes industrial cleaning electricity, full-scale media regeneration or continuous laboratory-to-production transfer.
Regional matching and climate–cost thresholds	New analyses based on existing records, addressing route selection and transport under feedstock-quality constraints.	Reports interface conditions, uncovered terms, route and transport comparisons, regional quality–flow matching, and reproducible conditional ranges.

Table S3 Sampling characterization and evidence availability

Evidence item	What the retained dataset supports	Availability and interpretation
Record linkage	60 sample IDs, enterprise IDs and campaign labels	Sampling_Characterization_Register.csv lists all IDs. Specific source lot, sampling position, increment number, reduction/compositing and confirmatory replicates are unavailable.
Wet and dry mass	Compiled wet and dry mass per sample	Regional drying conditions and sample-specific weighing sheets are unavailable. The PE 60 °C procedure is not transferred to these samples.
Polymer identity	Reported incoming-material information plus portable infrared procedure	Sample-specific spectra, library records, decision rules and ambiguous-material records are unavailable. Procedure reporting is not independent identification validation.
Non-plastic categories	Paper, mineral and oil-associated operational categories	An oil-specific validated assay and detection limits are unavailable. A recorded zero is not proof of absence.
Other impurities	Dry mass minus the seven explicitly listed masses	A calculated residual, not an independently weighed category. Closure cannot validate analytical recovery.
Decision rule	Unique classification of 60 records at 5.0%	At 4.5%, seven II-PE and two II-PP records move to U; at 5.5%, no change. This is rule sensitivity, not measurement uncertainty.
Technical applicability	Separate conservative evidence layer	Composition classes do not validate removable contamination, inlet compatibility or product function; unresolved mass is retained in technical U.
Physical intervention links	25 candidate handling-flow records	Intervention_Link_Register.csv records unavailable transaction/lot/node links. A verified non-overlapping subset and its coverage cannot be established.

PP, polypropylene.

Table S4. Principal symbols and units. The feasible allocation set Q, annual capacity Q_{cap} and qualification share q refer to distinct quantities.

Symbol	Definition	Unit or range
FU	PE pellet output or treatment of the same MIX feed	1 t pellets (retained under assumed q); 1 t MIX feed

Symbol	Definition	Unit or range
m_{wet}, m_{dry}, m_k	Sample wet, dry and component masses	g within each sample
w, c_{imp}, c_{PO}, c_{OP}, ε	Moisture, component percentages and closure difference	% in S1–S4; w fraction in S8
F_j, P_j, E_j	Batch feed, product and electricity	kg; kg; kWh
Y, $e_{pellets}$, e_D	Apparent output; pelletization and cleaning electricity	kg/kg; kWh/t pellets; kWh/t incoming dry-film equivalent
b, r_R, q_R, μ_R	Dry pelletization feed coefficient; recovery; assumed retained-output share; incoming requirement	t/t pellets; fractions; fractions; t/FU
A_{ri}, EF_i	Mapped activity and characterized factor	Compatible activity and reference units
G^{cov}, U, H^{cov}	Covered climate; unknown climate; covered climate at fixed distances	kg CO_2-eq/FU
$C^{L,cov}$, U_{cost}, $R_{product}$	Covered cost tier; unknown cost; product revenue	CNY/FU
I, S, i, n, Q_{annual}	Capital, residual value, discount, life and annual throughput	CNY; CNY; fraction; year; t/year
μ_R, d_R, d_f	Transport coefficient, route distance, recorded flow distance	t/FU; km; km
f_T, p_T, β_R, A, B	Freight factor/price, varied-distance coefficient, intercept and slope	kg CO_2-eq/(t·km); CNY/(t·km); t/FU; impact/FU; impact/(FU·km)
M_f, M_e, $x_{f,s}$	Flow, enterprise and allocated state mass	t
$\omega_{e,t}$, $\pi_{e,s}$, α	Campaign weight, annual state-share proxy and adoption	Fractions from 0 to 1
eligible_f, Q, a, b	Candidate flag, feasible set, process and distance coefficients	0/1; constrained set; per t feed; per t·km
η_{EB}, s_{EB}, e_D	EB product yield, EB electricity and dry-cleaning electricity	kg/kg feed; kWh/kg product; kWh/t dry-film equivalent
V, m_{FU}, I_{water}	Water supply, normalization mass and water intensity	m^3; t; m^3/t
Q_{cap}, L_{cap}, o, v	Output/supply caps and allocation coefficients	t/year; t/year; t output/t baseline feed; t extra/t baseline feed

FU, functional unit; EB, extrusion-based engineering reference.

Table S5. Route applicability and supporting evidence. Citation numbers follow the main text; complete source details are provided in S3_Route_Evidence.

Quality state	Candidate route	Applicability conditions	Evidence type	Specific source	Scope of support
I-PE	Re-pelletizing after sorting and size reduction	PE-dominated feed meeting target product requirements; case-specific pelletizer inlet moisture ≤1% and particle size 5–15 mm.	enterprise-derived record; engineering estimate; underlying record not independently verified	Pelletizing Equipment Parameters, rows 3–4; Demonstration Line Report (28 October 2025), Sections 1.3 and 3.2; S4.	Defines the inlet conditions for the case PE pelletizer and supports pellet output from the recorded feed; product requirements must be assessed for the corresponding formulation and application.
I-PP	Mechanical recycling after sorting	A single-PP stream with contamination, melt-processing conditions, and target products appropriately matched.	transferred inventory	[3], mechanical recycling and sorting.	Provides process evidence for mechanical recycling of single-PP streams for candidate-route screening.
II-PE	Re-pelletizing after dry cleaning	Low-moisture PE with surface contamination suitable for gas–solid cleaning; cleaned feed meets the case pelletizer inlet conditions.	enterprise-derived record; engineering estimate; transferred inventory; underlying record not independently verified	[42]; S4; dry-cleaning module in S6.	Laboratory gas–solid cleaning evidence supports apparatus use, not industrial electricity or media demand. Independent pelletization records are linked conditionally; preparation, media regeneration and product qualification remain explicit boundaries.
II-PE	Re-pelletizing after washing and drying	Washing targets removable surface contamination, with dewatering, drying, and wastewater treatment; feed meets pelletizer inlet and product requirements.	transferred inventory; engineering estimate	[26], Section 3.2 and Tables 2–3; S6.	Provides industrial PE washing, drying, and on-site water-treatment inputs for linking the wet-cleaning module.
II-PP	Mechanical recycling after suitable cleaning	Cleaning and drying are selected for the contamination characteristics of the PP stream, followed by checks against equipment and product requirements.	transferred inventory	[3], mechanical recycling and contamination.	Supports selection of PP pretreatment by resin and contamination characteristics for qualitative route screening.
III	Direct products by melt compounding and compression molding	Mixed feed meets the case feeding and compounding conditions; engineering documentation limits PET to 5%, with foreign-object removal, shredding, and homogenization.	enterprise-derived record; engineering estimate; underlying record not independently verified	Molded Pallet Equipment Parameters, rows 2–4; S5; pallet report ZTWB230228001-0001.	Seven cycles provide molding mass and electricity inputs; the same-feed comparison uses MIX-06/MP-06. Material specimens correspond to MIX-01, MIX-04, and MIX-07; pallet loads describe separately tested products.
III	Extrusion-based engineering reference (EB; not a measured MIX-06 trial)	Processable extrusion yields and electricity inputs are assumed for the same MIX feed; product function is assessed separately.	transferred inventory; analyst-defined scenario	[32], Table 5; S6 and S7.	Reference electricity includes motor and barrel heating in recycled polypropylene (rPP) extrusion. MIX conditioning, filtration, board forming, cooling, startup allocation and non-product treatment are not all quantified; product-service equivalence is unverified.

Quality state	Candidate route	Applicability conditions	Evidence type	Specific source	Scope of support
IV	Enhanced sorting or other treatment (outside quantitative comparison)	Mixed-polymer, contamination, and moisture conditions are first verified before assessing sorting and mechanical or chemical treatment feasibility.	transferred inventory	[3], mixed-feed and chemical recycling.	Supports qualitative screening of further treatment for highly mixed or contaminated feed; state IV remains outside the quantitative route comparison.

PET, polyethylene terephthalate.

Table S6. Sensitivity of record-based Class II allocation to the non-plastic-impurity upper bound and scope of the additional proxy analyses.

Metric	IMP4.5	IMP5.0	IMP5.5
Class II impurity upper bound (% dry basis)	4.5	5.0	5.5
II-PE sample count (*n*)	16	23	23
II-PE proxy mass (t)	6133.975	8761.838	8761.838
Equal-frequency candidate mass interval (t)	1756.807–6040.318	3490.943–7272.641	3490.943–7272.641
U sample count	9	0	0
U equal-frequency proxy mass (t)	2945.697	0	0
Annual-quality proxy families	10	10	10
Regional state count (including U)	7	7	7
Monthly-weighted candidate mass interval (t)	1467.465–5269.491	2728.697–6605.198	2728.697–6605.198

Candidate-mass intervals use the quality proxy named in each row over all 53 sampled-enterprise flows, with adoption one and without deployment caps. They are feasible allocation ranges, not observed diverted masses or statistical confidence intervals. Capacity-limited climate and cost endpoints depend additionally on the process coordinate, adoption, functional basis and supply condition and are reported in S8.

The full sample threshold distances and ten annual-quality proxies are generated from the regional inputs. Candidate mass means II-PE allocated to recorded washing-to-pellet flows, not all II-PE proxy mass. Regional endpoint files report the optimization objective, adopted mass, transport work, output, extra feed, capacity use and status together. Climate and cost differences use reference minus first route, and are optimized independently.

Table S7 PE batch mass and electricity account. SD, standard deviation.

Batch	Feed kg	Pellets kg	Solid kg	Difference kg	Yield %	kWh/t feed	kWh/t pellets
GR-01	3,099	2,884	138.0	77.0	93.062	394.321	423.717
GR-02	3,839	3,575	173.4	90.6	93.123	394.634	423.776
GR-03	3,475	3,266	126.6	82.4	93.986	411.511	437.844
GR-04	3,022	2,816	131.4	74.6	93.183	405.361	435.014
GR-05	3,637	3,421	114.2	101.8	94.061	414.627	440.807
GR-06	2,999	2,822	101.8	75.2	94.098	423.141	449.681
GR-07	3,401	3,201	110.4	89.6	94.119	426.345	452.983
GR-08	3,755	3,515	131.1	108.9	93.609	422.104	450.925
GR-09	3,078	2,887	108.0	83.0	93.795	413.580	440.942
GR-10	3,762	3,538	120.6	103.4	94.046	405.369	431.034
GR-11	2,927	2,730	112.5	84.5	93.270	420.567	450.916
GR-12	3,802	3,571	129.8	101.2	93.924	415.045	441.893

Metric	Mean	Sample SD	Minimum	Maximum	Pooled
Yield_percent	93.690	0.418	93.062	94.119	93.700
Electricity_kWh_per_t_pellets	439.961	10.199	423.717	452.983	439.779
Electricity_kWh_per_t_feed	412.217	10.557	394.321	426.345	412.075

SD uses n−1 and represents dispersion among retained records. Yield SD is in percentage points. The pooled inventory is a ratio of totals. Sources: batch IDs GR-01–GR-12 in S4 and process/inputs/pe_batches.csv. Total feed = 40,796 kg; pellets = 38,226 kg; recorded residue = 1497.8 kg; unallocated difference = 1072.2 kg. The non-pellet 6.299637% comprises 3.671438% recorded residue and 2.628199% difference. Assigning the 299.524 kg inlet gravimetric diagnostic entirely to the difference leaves 772.676 kg; neither quantity identifies a measured emission.

Table S8 Batch influence and reporting resolution scenarios

Omitted batch	Pooled electricity kWh/t pellets	Pooled yield %
GR-01	441.090	93.753
GR-02	441.430	93.760
GR-03	439.960	93.674
GR-04	440.158	93.742
GR-05	439.678	93.665
GR-06	438.990	93.669
GR-07	438.572	93.662
GR-08	438.651	93.710
GR-09	439.684	93.693
GR-10	440.671	93.665
GR-11	438.923	93.734
GR-12	439.561	93.677

Scenario	Electricity envelope	Climate root km	L2 root km
PE nearest integer E and mass	439.553–440.005 kWh/t pellets	Not used	Not used
PE truncated E; rounded mass	439.710–440.162 kWh/t pellets	Not used	Not used
MIX rounded E; mass to 0.1 kg	267.717–300.158 kWh/t feed	61.409–239.357	11.833–56.170
MIX truncated E; rounded mass	283.465–315.956 kWh/t feed	−25.246–152.976	−9.757–34.647

Nearest-integer scenarios use ±0.5 of the reported unit; truncated electricity uses [E,E+1), with reported upper endpoints interpreted as limiting values. The historical rounding rule and meter accuracy are unverified. These are alternative reporting-rule envelopes, not instrument error estimates or confidence intervals. Nonpositive roots retain their sign and indicate no positive advantage-loss crossing from the stated origin. Leave-one-batch-out results span 438.572–441.430 kWh/t pellets and describe influence of individual retained records, not population uncertainty.

Table S9 Characterized background factors and service scope

The retained factor snapshot uses ecoinvent 3.10, allocation cut-off by classification, system processes, SimaPro 10.1.0.4 and the Intergovernmental Panel on Climate Change (IPCC) 2021 100-year global warming potential (GWP100) method [49], V1.03 [50]. The recorded snapshot export is 9 September 2026, with database/method version supported by later interface verification; export date is not a data representative year. Data representative years and separate freight load/empty-return parameters are unavailable. Values below are already converted to the reported activity unit; the MJ-to-kWh multiplier is applied only once. Universally unique identifiers (UUIDs), full names, reference products and revisions are reproduced in Background_Factors_18.csv. Its separate data_representative_year field marks each unavailable year as not reported in the supplied factor snapshot.

ID	Process and UUID	Geography	Reference product / activity unit	Dataset revision
25543	Electricity, medium voltage {CN-ECGC}| market for electricity, medium voltage | Cut-off, S d13f1c44-ed61-501d-93d7-001606e1ea94	CN-ECGC	Electricity, medium voltage / kWh	1.3.0.0
25280	Electricity, low voltage {CN-ECGC}| market for electricity, low voltage | Cut-off, S 1bf9c072-3c65-5224-9354-0ed9eb8b432b	CN-ECGC	Electricity, low voltage / kWh	1.3.0.0
25561	Electricity, medium voltage {CN}| market group for electricity, medium voltage | Cut-off, S 3f5e5da4-dd29-5d57-81db-306a02ee054d	CN	Electricity, medium voltage / kWh	1.3.1.0
25269	Electricity, low voltage {CN}| market group for electricity, low voltage | Cut-off, S 61ccb666-1b5f-5392-acea-5b737a356240	CN	Electricity, low voltage / kWh	1.3.1.0
20173	Tap water {RoW}| market for tap water | Cut-off, S 9c5a283f-37d0-5818-9e42-0484c5027ef6	RoW	Tap water / kg	3.3.0.0
20179	Tap water {GLO}| market group for tap water | Cut-off, S c0e5d79b-8792-59ac-8198-47d25ce19356	GLO	Tap water / kg	1.3.1.0
20277	Water, deionised {RoW}| market for water, deionised | Cut-off, S 03a264ae-20c0-5e58-a33b-bc613dc10a7b	RoW	Water, deionised / kg	3.3.1.0
12380	Polyaluminium chloride {GLO}| market for polyaluminium chloride | Cut-off, S 6ea129ff-f2ce-5367-a6ef-c1d0baa20341	GLO	Polyaluminium chloride / kg	4.3.0.0
13307	Polyacrylamide {GLO}| market for polyacrylamide | Cut-off, S 27e7a77a-d170-50c0-a428-20628feb5e09	GLO	Polyacrylamide / kg	5.3.0.0

ID	Process and UUID	Geography	Reference product / activity unit	Dataset revision
12497	Sodium hydroxide, without water, in 50% solution state {RoW}| market for sodium hydroxide, without water, in 50% solution state | Cut-off, S 3f7c9087-db3d-56e7-8839-d521ca58a834	RoW	Sodium hydroxide, without water, in 50% solution state / kg	9.3.0.0
12430	Silica sand {GLO}| market for silica sand | Cut-off, S ab9f329a-709a-5c36-8a0a-00a5f491e49f	GLO	Silica sand / kg	4.3.1.0
12947	Silica sand {RoW}| silica sand production | Cut-off, S 71c129fc-080b-5348-b616-95f56653dabc	RoW	Silica sand / kg	99.3.0.0
28198	Transport, freight, lorry, unspecified {RoW}| market for transport, freight, lorry, unspecified | Cut-off, S 53405279-b24f-53ae-950d-748a7af93e3a	RoW	Transport, freight, lorry, unspecified / tkm	2.3.0.0
28224	Transport, freight, lorry 16-32 metric ton, EURO5 {RoW}| market for transport, freight, lorry 16-32 metric ton, EURO5 | Cut-off, S ffe453ac-507c-5345-a800-5520b3831aa9	RoW	Transport, freight, lorry 16-32 metric ton, EURO5 / tkm	3.3.1.0
28230	Transport, freight, lorry 16-32 metric ton, EURO6 {RoW}| market for transport, freight, lorry 16-32 metric ton, EURO6 | Cut-off, S 5b59f702-6998-5c11-ab22-0dee73db9816	RoW	Transport, freight, lorry 16-32 metric ton, EURO6 / tkm	2.3.0.0
28353	Transport, freight, lorry >32 metric ton, EURO5 {RoW}| market for transport, freight, lorry >32 metric ton, EURO5 | Cut-off, S 5dc88152-5b40-56ff-b21d-5d792b95adee	RoW	Transport, freight, lorry >32 metric ton, EURO5 / tkm	3.3.1.0
36115	Wastewater, average {RoW}| market for wastewater, average | Cut-off, S 93b25302-6739-5955-be56-d807f265bea2	RoW	Wastewater, average / m^3	3.3.0.0
36198	Wastewater, average {RoW}| treatment of wastewater, average, wastewater treatment | Cut-off, S 050d4d84-20dc-53d4-9454-0c93b7c507d0	RoW	Wastewater, average / m^3	6.3.0.0

ID	Native unit	Multiplier	Fossil	Biogenic	Land change	Total kg CO_2-eq/unit
25543	MJ	3.6	0.856203460	0.000144718	0.000459062	0.856807240
25280	MJ	3.6	0.883194186	0.000161669	0.000478978	0.883834832
25561	MJ	3.6	0.949804210	0.000123869	0.000326828	0.950254906
25269	MJ	3.6	0.961618207	0.000141650	0.000342806	0.962102663
20173	kg	1	0.001276188	0.000001047	0.000001049	0.001278284

ID	Native unit	Multiplier	Fossil	Biogenic	Land change	Total kg CO_2-eq/unit
20179	kg	1	0.000878506	0.000001040	0.000001053	0.000880598
20277	kg	1	0.000462770	0.000034391	0.000000447	0.000497607
12380	kg	1	1.816745177	0.000960763	0.001384061	1.819090001
13307	kg	1	3.995947145	0.001523162	0.001700499	3.999170806
12497	kg	1	1.488066765	0.001161348	0.001682847	1.490910961
12430	kg	1	0.043118098	0.000013586	0.000035600	0.043167284
12947	kg	1	0.035430191	0.000011888	0.000017791	0.035459869
28198	tkm	1	0.156113864	0.000025057	0.000063672	0.156202593
28224	tkm	1	0.195638112	0.000030194	0.000077623	0.195745929
28230	tkm	1	0.191650614	0.000030257	0.000077846	0.191758717
28353	tkm	1	0.107814683	0.000017326	0.000043149	0.107875157
36115	m^3	1	0.261593600	0.030256632	0.000235377	0.292085609
36198	m^3	1	0.411453860	0.073181629	0.000436515	0.485072004

The central electricity factor is ID 25543, 0.856807240395630 kg CO_2-eq/kWh; freight is ID 28198, 0.156202592699214 kg CO_2-eq/(t·km). Selected_Route_Component_Ledger.csv gives every matched activity × factor contribution and its subtotal. No mixed PE-residue incineration factor is supplied by this snapshot. A known waste destination does not imply zero climate burden. Shared terms cancel only with identical quantities and functional bases, including q. MP denotes the molding route; rPP denotes recycled polypropylene.

Service	PE dry / wet	MIX MP / EB
Cleaning and preparation	Dry electricity and recovery are analytical coordinates; wet washing is transferred inventory. Unmetered preparation is unknown.	MP cycle covers stated processing; EB is a transferred rPP extrusion parameter. Additional conditioning remains unknown.
Drying	Wet drying retained: local pelletization excludes it. Industrial dry cleaning/drying demand is not measured.	Unrecorded preparation is not silently included.
Pelletization or compounding	Common documented pelletization module, scaled by the applicable q.	MP compounding/heating within cycle; EB motor and barrel heating parameter.
Forming cooling startup	PE auxiliaries and batch preheating/standby within meter; cleaning/drying excluded.	MP pressing/cooling within the recorded cycle included; startup preheat excluded; additional services outside the cycle unquantified. EB complete board forming/cooling and startup not established.

Service	PE dry / wet	MIX MP / EB
Water and dosing	PE module make-up 0.260294 m³/t pellets; wet ordinary water 2.260294 plus 0.12663 m³ dosing water.	MP 0.060 and EB 0.080 m³/t are scenario inputs.
Chemicals and effluent	Transferred wet chemicals; on-site then off-site treatment in sequence. Missing charges explicit.	Auxiliary water/effluent scenarios; incomplete EB services explicit.
Residue and media	Known residue fee once; climate factor absent. Industrial sand loss/regeneration unknown.	Zero-price external trim/reject transfer; no credit; downstream burden unknown.
Transport and capital	Separate transport; conditional case capital and output capacity.	Same-feed transport and procurement cancel only under stated equal conditions; scenario capital.

Only climate change is characterized. Human toxicity, ecotoxicity, additive migration, product safety and water scarcity are not evaluated. PE wet-route matched freshwater input is approximately 2.386924 m³/t pellets; the PE module alone is 0.260294 m³/t. Because industrial dry-cleaning water is unquantified, the latter is not a complete dry-route total or a basis for a full-route water-saving percentage. Wastewater output, withdrawal and water consumption are not interchangeable.

Table S10. Services not fully covered and their treatment in route comparisons.

Item not fully covered	Treatment in this study
PE residues	Combined measured residue is externally incinerated; composition and an applicable climate factor are unavailable. CNY 400/t includes collection, loading and incineration. Climate burden remains unknown, not zero.
MIX residues and trimmings	Measured trim and rejects leave the cycle for free external recycling. There is no revenue, treatment charge or displacement credit. Downstream climate burdens and EB non-product fate remain unresolved.
PE product qualification	The twelve batches contain no newly added additives or masterbatch. An assumed retained-output share *q* scales activities, feed and transport per qualifying tonne; it is not an observed customer acceptance rate.
Media regeneration and other pretreatment	Industrial sand loss, regeneration and unresolved preparation remain unknown. Laboratory reuse is not an industrial allocation factor. Burden allowances identify conditional equalities, not proven service feasibility.
Wet-route chemicals and wastewater	NaOH uses 12 kg active substance from 24 kg of 50% solution; 126.63 kg dosing water is separate. On-site treatment and off-site effluent treatment are sequential. Chemical formulation and effluent proxies are not fully matched.
Deionized-water purchasing and wastewater charges	Unpriced deionized water and off-site settlements without cost breakdowns remain unknown costs. All-inclusive wastewater prices are not added to overlapping services.

Table S11 Cost coverage procurement and omitted cost allowances

L0 covers priced direct inputs and known PE residue treatment; L1 adds labor and maintenance; L2 adds annualized equipment. Procurement, transport, separately priced ancillary services and revenue remain explicit extensions. Unknown quantities or prices are not assigned zero. Full activity, unit, price, source status, billing scope, tier and missing-reason fields are in Cost_Coverage_Register.csv and the economic workbook. The register distinguishes unavailable quantities from unavailable prices, and identifies bundled charges and separately tested scenarios.

Cost group	Current treatment	Unpriced or unmatched scope
Direct inputs	Electricity and matched water/material inputs; PE residue fee 400 CNY/t	Deionized-water purchasing and effluent settlement are not completely priced.

Cost group	Current treatment	Unpriced or unmatched scope
Labor and maintenance	Case scenario charges added in L1	Original itemized transaction records unavailable; no inference of complete operating cost.
Annualized equipment	Case investments; 10 years, 6%, 5% residual; utilization once	Quotation scope equivalence and original dates not independently established; 2026 is a reference scenario.
Feed procurement	$\mu_R\, p_{\text{feed},R}$ added explicitly in S12a	Same-feed, same-price MIX terms cancel only in the difference. PE feed coefficients generally differ.
Transport	Segment quantity × distance × scenario tariff	Return settlement not separately evidenced; no duplicate bundled residue transport fee.
Auxiliary services	Priced amounts only when quantity and scope are known	Industrial sand loss/regeneration, some preparation and EB board forming/cooling remain unpriced.

p_W CNY/t	ΔC at δp=0 CNY/t	Dry-price premium at equality CNY/t
0	326.921	302.420
500	324.177	299.882
1000	321.433	297.344
2000	315.946	292.268
4000	304.971	282.115
6000	293.997	271.963

Illustration: e_D = 100 kWh/t dry cleaning feed, r_D = 0.98, r_W = 0.985, q_D = q_W = 1 and no assigned net unpriced cost. p_D = p_W + δp uses the same dry-film-equivalent mass basis. The scan uses p_W = 0, 500, 1000, 2000, 4000, 6000 and δp = −500, −250, 0, 250, 500 CNY/t, excluding negative p_D. These are specified scenarios, not market observations. Procurement_Stress_All_PE_Coordinates.csv repeats the 28 valid price pairs over all 252 PE engineering/q coordinates (7056 combinations). Each covered-plus-procurement difference is its own allowable net unpriced dry-route cost; a negative margin is not reported as a positive allowance.

At the MIX reference, an MP-minus-EB net unpriced cost of 20.421804 CNY/t feed removes the covered L2 advantage before any extra transport. PE residue contributes 15.673102 CNY/t pellets and is already counted. The chemical-price scenario uses 32.37 kg of commercial products × 2.60 CNY/kg = 84.162 CNY/t pellets, including 24 kg of 50% NaOH solution; purchasing is not priced at the 12 kg active-ingredient mass. Revenue scenarios remain separate from L2.

Table S12. Properties of three material-specimen groups with established feedstock links. Values are group means, with n = 5 for each property; formulations differ among groups.

Specimen group	Linked feed	n	Tensile strength / MPa	Flexural strength / MPa	Flexural modulus / MPa
EX-M1	MIX-01	5	20.736	23.873	1050.0
EX-M2	MIX-04	5	19.551	25.256	1239.8
EX-M3	MIX-07	5	18.550	27.333	1393.6
